# From fibre to function: are we accurately representing muscle architecture and performance?

**DOI:** 10.1111/brv.12856

**Published:** 2022-04-07

**Authors:** James Charles, Roger Kissane, Tatjana Hoehfurtner, Karl T. Bates

**Affiliations:** ^1^ Structure and Motion Lab, Comparative Biomedical Sciences Royal Veterinary College Hawkshead Lane Hatfield Hertfordshire AL9 7TA U.K.; ^2^ Department of Musculoskeletal & Ageing Science, Institute of Life Course & Medical Sciences University of Liverpool The William Henry Duncan Building, 6 West Derby Street Liverpool L7 8TX U.K.; ^3^ School of Life Sciences University of Lincoln, Joseph Banks Laboratories Green Lane Lincoln LN6 7DL U.K.

**Keywords:** biomechanics, muscle, functional morphology, locomotion, modelling, physiology

## Abstract

The size and arrangement of fibres play a determinate role in the kinetic and energetic performance of muscles. Extrapolations between fibre architecture and performance underpin our understanding of how muscles function and how they are adapted to power specific motions within and across species. Here we provide a synopsis of how this ‘fibre to function’ paradigm has been applied to understand muscle design, performance and adaptation in animals. Our review highlights the widespread application of the fibre to function paradigm across a diverse breadth of biological disciplines but also reveals a potential and highly prevalent limitation running through past studies. Specifically, we find that quantification of muscle architectural properties is almost universally based on an extremely small number of fibre measurements. Despite the volume of research into muscle properties, across a diverse breadth of research disciplines, the fundamental assumption that a small proportion of fibre measurements can accurately represent the architectural properties of a muscle has never been quantitatively tested. Subsequently, we use a combination of medical imaging, statistical analysis, and physics‐based computer simulation to address this issue for the first time. By combining diffusion tensor imaging (DTI) and deterministic fibre tractography we generated a large number of fibre measurements (>3000) rapidly for individual human lower limb muscles. Through statistical subsampling simulations of these measurements, we demonstrate that analysing a small number of fibres (*n* < 25) typically used in previous studies may lead to extremely large errors in the characterisation of overall muscle architectural properties such as mean fibre length and physiological cross‐sectional area. Through dynamic musculoskeletal simulations of human walking and jumping, we demonstrate that recovered errors in fibre architecture characterisation have significant implications for quantitative predictions of *in‐vivo* dynamics and muscle fibre function within a species. Furthermore, by applying data‐subsampling simulations to comparisons of muscle function in humans and chimpanzees, we demonstrate that error magnitudes significantly impact both qualitative and quantitative assessment of muscle specialisation, potentially generating highly erroneous conclusions about the absolute and relative adaption of muscles across species and evolutionary transitions. Our findings have profound implications for how a broad diversity of research fields quantify muscle architecture and interpret muscle function.

## I INTRODUCTION

**Fig. 1:**
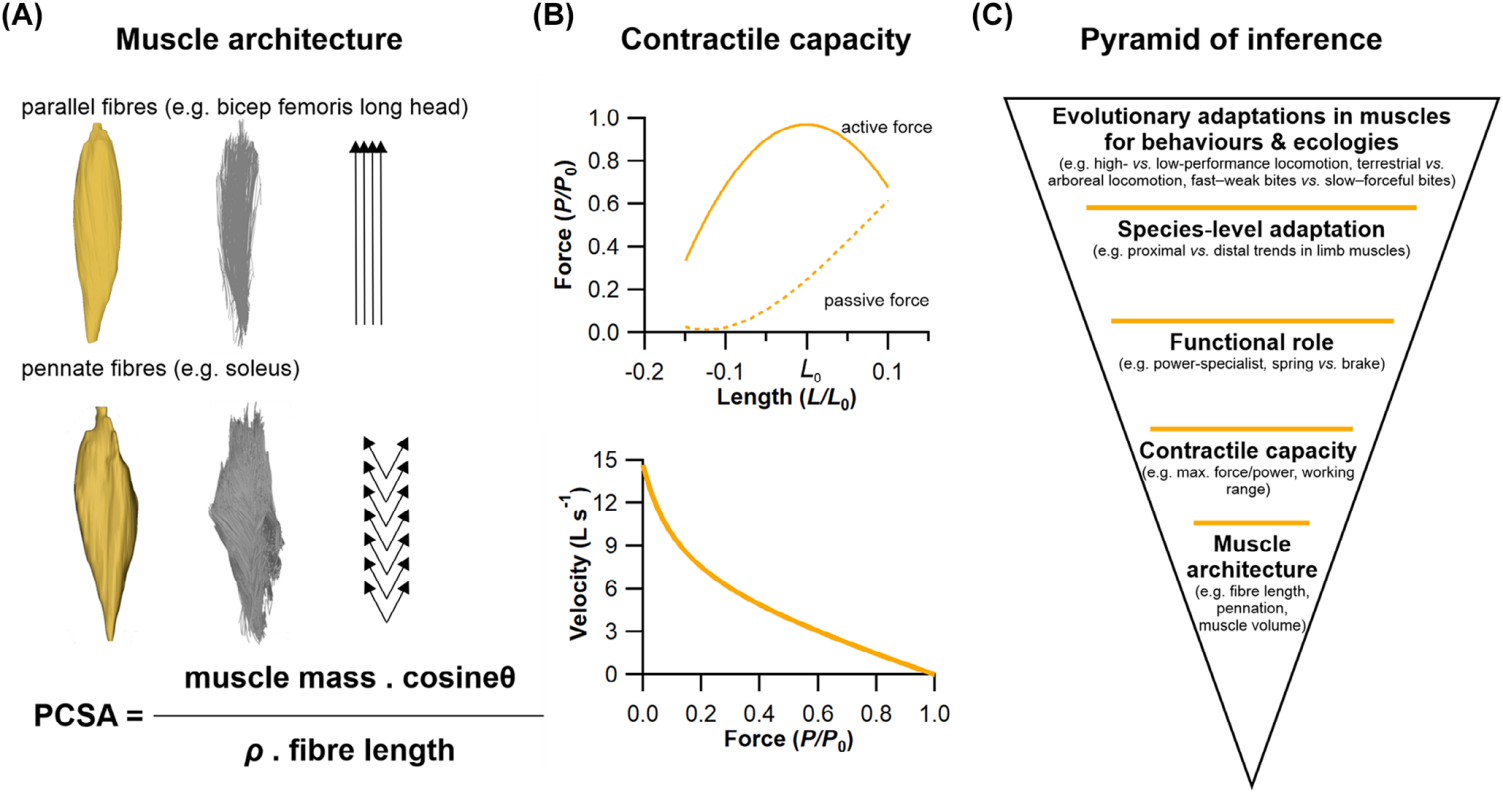
The theoretical relationships between muscle fibre architecture, contractile capacity and functional inferences. (A) The architecture of skeletal muscle, also known as the arrangement of a muscle's fibres in relation to its axis of force generation, can be broadly classed as either parallel fibred, with long fibres and little to no pennation angle (θ) or internal tendon/aponeurosis, or pennate, with shorter fibres orientated at an angle to an internal aponeurosis. This architecture can have a substantial impact on a muscle's ability to produce force, which is primarily determined by its physiological cross‐sectional area (PCSA), the formulation of which depends on a muscle's mass, fibre length and density (ρ; which is considered relatively homogeneous in skeletal muscle). (B) These crucial architectural parameters have a large impact on a muscle's contractile capacity, often quantified by its force–length or force–velocity relationships, where *L* = length, *L*_0_ = optimal length, *P* = force and *P*_0_ = optimal force. (C) This forms the foundation of a pyramid of inference, similar to that of Witmer (1995), of muscle fibre to function, upon which predictions or observations of how a muscle functions in a dynamic context are subsequently used to infer species‐level adaptations in a muscle's function, and finally to generate hypotheses surrounding potential adaptations of a muscle across species spanning major evolutionary and/or ecological transitions.

Almost all animal motion is powered by striated skeletal muscle (Alexander, 2003). Skeletal muscle consists of cells known as fibres, which are bound in fascicle bundles usually considered functionally equivalent to a single muscle fibre (Bodine *et al*., 1982). Since the seminal work of Nobel laureate A.V. Hill in the 1930s (Hill, 1938) it has been recognised that the macroscopic size and arrangement of these fibres, commonly collectively defined as muscle architecture (e.g. Lieber & Fridén, 2000), play a pivotal role in determining how a muscle functions. This functional capacity can be simplistically quantified using data on gross fibre properties through the calculation of its physiological cross‐sectional area (PCSA), which is directly proportional to a muscle's maximum force‐generating capacity (Lieber & Fridén, 2000) (Fig. 1A), as well as through more complex studies into relationships between the contractile force, length and velocities of its fibres (Luff, 1981) (Fig. 1B). With regard to the macroscopic arrangement of muscle fibres, different muscle structures exist that can directly impact how they function. For instance, fibres can, on a basic level, run in parallel from the origin of a muscle to its insertion (i.e. parallel fibred), or at an angle to the muscle's line of action and attach to an internal tendon or aponeurosis (i.e. pennate fibred) (Fig. 1A). Parallel‐fibred muscles, often with long fibre lengths, are thought to be adapted to produce high‐velocity contractions, while muscles with shorter and more pennate fibres are adapted for higher force output by virtue of their greater fibre number (Fig. 1A). As such, the length of these fibres, along with their pennation angle and mass of the muscle belly, are crucial factors to measure accurately when assessing how a muscle functions during a dynamic movement.

The adaptation of skeletal muscle to function in a particular physiological niche is a complex process where remodelling may occur across the entire motor unit [i.e. changes in motor unit firing patterns (Sharples & Miles, 2021), in neuromuscular junction function (Padilla *et al*., 2021), the muscle metabolome (O'Brien *et al*., 2021) and in fibre architecture (Taylor *et al*., 2009)], any or all of which can affect motor function. The mechanisms behind the temporal changes that occur across the central and peripheral nervous systems are much debated, however, it is logical to hypothesise that the varying functional properties afforded by different muscle architectures have been exploited to generate adaptations in muscles to power distinct motions and behaviours. This expectation has led researchers from research fields as diverse physiology (Luff, 1981; De Ruiter, De Haan & Sargeant, 1995; Talmadge *et al*., 2002), zoology (Payne *et al*., 2005a; Williams, Payne & Wilson, 2007a; Eng *et al*., 2008; Williams *et al*., 2008b; Allen *et al*., 2010; Paxton *et al*., 2010, 2014; Wareing *et al*., 2011; Hudson *et al*., 2011a,b; Lamas, Main & Hutchinson, 2014; Charles *et al*., 2016a; Rose *et al*., 2016a; Rose, Nudds & Codd, 2016b), anthropology (Payne *et al*., 2006; Myatt, Crompton & Thorpe, 2011; Myatt *et al*., 2012; O'Neill *et al*., 2013), clinical and veterinary biomechanics (Steele *et al*., 2010; Rankin, Rubenson & Hutchinson, 2016; Charles, Cappellari & Hutchinson, 2018; Ellis, Rankin & Hutchinson, 2018; Stark *et al*., 2021), dentistry (Langenbach & Weijs, 1990), ageing research (Wilkinson, Piasecki & Atherton, 2018), sports and exercise science (Gonzales *et al*., 2019), biomimetic robotics (Jenkins & Bryant, 2019), and palaeontology (Bates & Schachner, 2012; Bates & Falkingham, 2018) to attempt to quantify the fibre architecture of muscles accurately to understand how they function at the most fundamental level (Luff, 1981; De Ruiter *et al*., 1995; Talmadge *et al*., 2002), how they are adapted to power key motions and behaviours that underpin the exploitation of specific ecological niches (Payne *et al*., 2005a; Carlson, 2006; Myatt *et al*., 2011; Hudson *et al*., 2011a,b; Bates & Schachner, 2012; Charles *et al*., 2016a; Bates & Falkingham, 2018), and how muscle performance can be maintained or improved in disease, dysfunction and sporting contexts (Steele *et al*., 2010; Wilkinson *et al*., 2018; Gonzales *et al*., 2019). This ‘fibre to function’ paradigm can be conceptualised as a pyramid of inference, similar to that of Witmer (1995), where higher‐level interpretations based on the interaction between muscle design and function rest upon the accurate founding characterisation of fibre architecture (Fig. 1C).

In this review, we provide a synopsis of how this fibre to function paradigm has been applied to understand muscle design, performance and adaptation in animals (Fig. 1C). We also review the different methods used in past studies to measure muscle architecture, with a specific focus on muscle fibre lengths, and briefly discuss the advantages and limitations of each method. As well as highlighting the widespread application of the fibre to function paradigm across a diverse breadth of biological disciplines, our review highlights a potential and highly prevalent limitation running through past studies. Specifically, we find that quantification of muscle architectural properties (fibre lengths, pennation angles and subsequently PCSA) is almost universally based on an extremely small number of fibre measurements. Despite the volume of research into muscle properties, across a diverse breadth of research disciplines, the fundamental assumption that a small proportion of fibre measurements can accurately represent the architectural properties of a muscle has never been quantitatively tested. It therefore remains unknown whether potential inaccuracies in the representation of architectural properties derived from inadequate sample sizes impacts higher‐level interpretations of basic muscle function and how muscles are (or are not) adapted to power specific motions within and across species (Fig. 1C). Herein, we subsequently apply a novel combination of state‐of‐the‐art medical imaging, image and statistical analysis, and physics‐based computer simulation of walking and jumping to address these fundamental issues for the first time. Our findings have profound implications for how a broad diversity of research fields quantify muscle architecture and utilise the fibre to function paradigm to interpret adaptive patterns in muscle function.

## II BACKGROUND

### (1) The fibre to function paradigm

As noted above, herein we adopt the definition of muscle architecture of Lieber & Fridén (2000, p. 1647), who described it as the “macroscopic arrangement of muscle fibres.” Measurements of muscle architecture, specifically fibre lengths, pennation angles and PCSA, are widely used across a range of biological disciplines to understand functional morphology at different scales. Here we attempt to provide a concise overview of the range of analyses carried out across this extremely broad diversity of research fields by dividing the literature into four artificial but convenient categories. In particular, these categories attempt to capture the diversity of research at different levels of the fibre to function pyramid of inference (Fig. 1C) starting with smaller‐scale analyses of the basic contractile behaviour of muscles (‘muscle physiology’), followed by comparative studies that seek to quantify adaptive links between muscle architecture across muscles within (‘musculoskeletal modelling’) and across (‘comparative anatomy and zoology’) species, and finally high‐level analyses of evolutionary adaptations in muscles that may represent key innovations underpinning the exploitation of specific behaviours or ecologies (‘evolutionary biomechanics’).

#### (a) Muscle physiology

Measures of muscle architecture played a key role in determining some of the fundamental or qualitative contractile behaviours of muscles (Fig. 1A, B). At the base of the fibre to function pyramid of inference (Fig. 1C) it has been commonplace for measures of muscle architecture to be combined with other data (e.g. *in‐vivo* contractile behaviour) to provide detailed assessments of the physiological and mechanical performance of muscles. In human muscles, for instance, Wickiewicz *et al*. (1984) tested a commonly used model of muscle dynamics (Hill, 1938) by relating the torque produced by specific lower limb muscle groups to the contraction velocity at which these torques were produced. The experimental data showed a close match to the theoretical predictions, and the relationships between their measurements of fibre length and PCSA and these torque–velocity relationships indicated that these architectural parameters have a substantial influence on muscle contractile dynamics (Wickiewicz *et al*., 1984).

Beyond this and a few other studies in humans (Barrett, 1962; Reeves & Narici, 2003), the majority of work into the relationships between muscle architecture, physiology and contractile capacity has been carried out in *ex vivo* experiments of rodent hindlimb muscles (Close, 1969; Baker & Hall‐Craggs, 1978; Crow & Kushmerick, 1982; Roy *et al*., 1985; De Ruiter *et al*., 1995; James, Altringham & Goldspink, 1995; Askew & Marsh, 1997; Kissane, Egginton & Askew, 2018), and to a lesser extent, other animals (Rack & Westbury, 1969; Close, 1972; Loeb *et al*., 1987; Lombardi & Piazzesi, 1990; Lieber & Brown, 1992; Pate *et al*., 1995; Pellegrino *et al*., 2003; Butcher *et al*., 2010). For example, studies into the soleus (SOL) and extensor digitorum longus (EDL) muscles of the mouse found that power output can be increased by increasing the duration of shortening instead of increasing contractile velocity (Askew & Marsh, 1997) and that the cost of maintaining a tetanic contraction within fast‐twitch muscle fibres is three times greater than in slow‐twitch fibres, although this difference decreases to around 50% after 12 s of stimulation (Crow & Kushmerick, 1982). In rat muscles, Kissane *et al*. (2018) found a regional variation in mechanical performance and resistance fatigue in the EDL, suggesting that a differential recruitment pattern of these regions is possible during locomotion, while Roy *et al*. (1985) showed that overstimulation of the ankle plantarflexor muscles leads to increased muscle mass and proportion of slow‐twitch muscle fibres, however these responses are ultimately governed by regional variation in fibre type and structure. Measures of muscle architectural properties were a key component of these analyses of contractile behaviour.

In other animals, Butcher *et al*. (2010) found strong relationships between the *in vivo* contractile behaviour of both the deep and superficial digital flexor of horses and their architecture (fibre lengths and pennation angle) and physiology and suggested that architecture may in fact have a greater influence on muscle function during locomotion than, for example, fibre type. Additionally, Lieber & Brown (1992) and Lieber & Shoemaker (1992) studied the relationships between sarcomere length and joint angles in the hindlimb muscles of frogs, which reflect the interaction between fibre length (or sarcomere number) and muscle moment arm. They found a high degree of variability in fibre length to moment arm ratio amongst the studied muscles, even within functional groups, suggesting that the lengths at which muscles produce their optimal force, and therefore their relative contribution to overall joint torques, is disparate even within muscles with a similar functional role.

Overall, these studies on isolated fibres or muscles, which rely heavily on the accurate characterisation of muscle architecture, have collectively provided a link between architecture, physiology and function which has underpinned subsequent interpretations and predictions of muscle function on whole‐system, within‐species as well as cross‐species scales.

#### (b) Musculoskeletal modelling

Moving up the fibre to function pyramid of inference (Fig. 1C), musculoskeletal models are widely used to integrate measures of the architecture of individual muscles with experimental motion data to assess variations in muscle function and their absolute and relative contribution to a particular behaviour or movement task. In other words, such approaches examine the varying functional roles of muscles within a species and how disparate architectures within a structure (e.g. a limb) might facilitate efficient motion. These models provide a unique means to predict dynamic muscle functional parameters that are hard or often impossible to measure with purely experimental methods (Damsgaard *et al*., 2006; Seth *et al*., 2018).

Musculoskeletal models are computational representations of bone geometries, joint morphologies, musculotendon unit (MTU) attachments and force‐generating properties. In human biomechanics, these models have traditionally been generic (constructed by merging anatomical data from multiple individuals, often cadavers) and subsequently scaled to match the anthropometry of a certain individual or participant in an experimental gait study (Arnold *et al*., 2010; Rajagopal *et al*., 2016). Such models have been used in clinical contexts to, for example, predict the muscle forces involved in crouch gait in children with cerebral palsy (Steele *et al*., 2012), to investigate contact forces at the knee in osteoarthritic patients (Richards *et al*., 2018) and to optimise the design of lower limb assistive devices such as prosthetics and exoskeletons through the prediction of muscle activation patterns and functions (Grabke, Masani & Andrysek, 2019). In sporting contexts, the mechanisms behind injuries (Bulat *et al*., 2019), individual muscle mechanics during sprinting (Schache *et al*., 2012) and the optimisation of strength training regimes (Plüss *et al*., 2018) have all been studied with these generic musculoskeletal models.

However, recent bodies of work have begun to develop optimised frameworks for developing accurate subject‐specific models, where medical imaging techniques such as magnetic resonance imaging (MRI) or computed tomography (CT) are used in combination with model building tools such as NMSBuilder (Valente *et al*., 2017) to create models containing the musculoskeletal geometry and MTU force‐generating properties from a specific individual (Charles *et al*., 2020). These models have been used to generate personalised predictions of anterior cruciate ligament forces during walking (Charles, Fu & Anderst, 2021) and to investigate knee contact forces after total knee arthroplasty (Hosseini Nasab *et al*., 2020).

Musculoskeletal models have also become a valuable tool to predict muscle dynamic behaviour in various animal species. These models can provide valuable insights into the muscle dynamics of species that are too small, large or rare to study extensively with *in vivo* methods. For instance, models of a mouse (Charles *et al*., 2018) and ostrich (*Struthio camelus*) (Rankin *et al*., 2016) constructed through medical imaging and manual dissection experiments to gather muscle architecture data (fibre lengths, pennation angles, and PCSAs), predicted that the lower limbs of small non‐cursorial mammals may not possess the muscle adaptations for energetically efficient locomotion seen in larger vertebrates (i.e. a proximo‐distal gradient of muscle function; Charles *et al*., 2018), while larger straighter limbed species likely make greater use of elastic energy storage in the distal hindlimb tendons to optimise locomotor economy (Rankin *et al*., 2016). Additionally, more complex predictive simulations have been used to predict the potential jumping performance of the tinamou (*Eudromia elegans*) (Bishop *et al*., 2021a). This work suggested that the strength of the distal lower limb muscles in this generalised bird are crucial in optimising jump height. Similar models have been used to examine muscular contributions to maximum bite force in a diverse array of animals (Curtis *et al*., 2010; Bates *et al*., 2010b; Bates & Falkingham, 2012; Watson *et al*., 2014; Broyde *et al*., 2021).

**Fig. 2:**
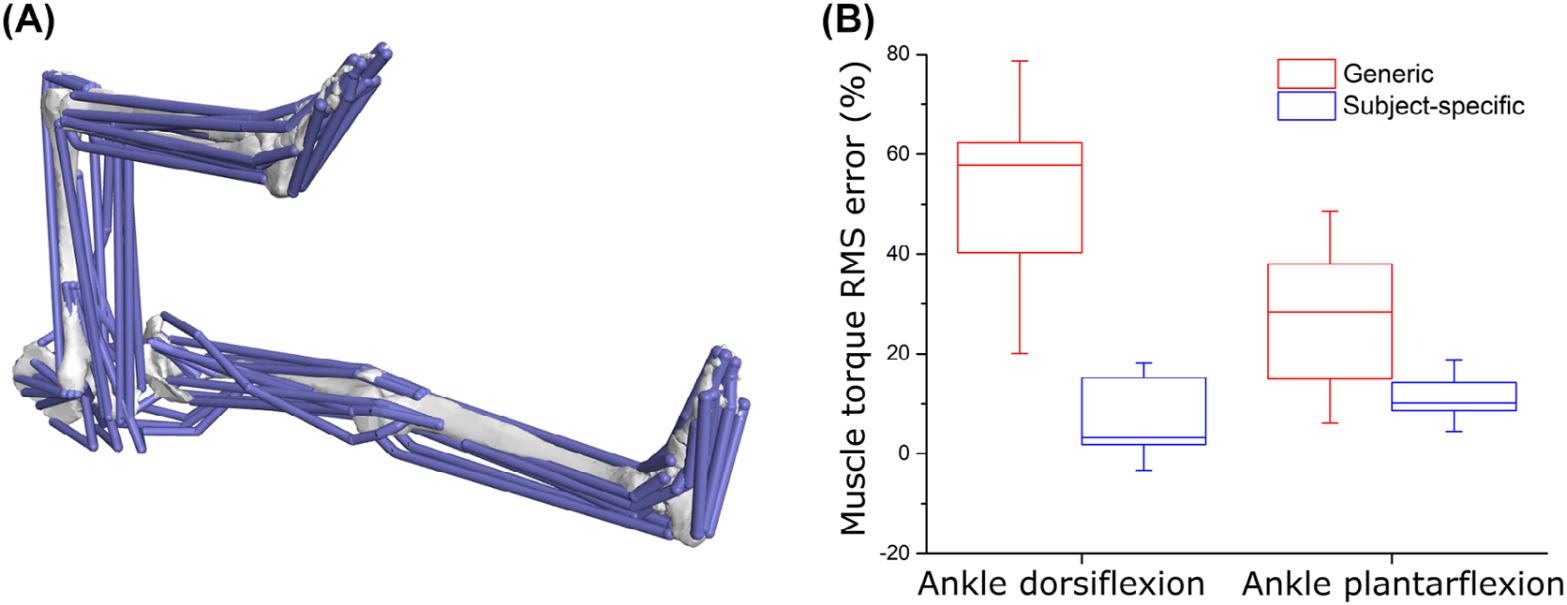
The advantages of subject‐specific muscle architecture for predicting muscle functional performance. (A) In a recent study, Charles *et al*. (2020) created 10 subject‐specific lower limb musculoskeletal models which included individualised muscle architecture data obtained from medical imaging. Here, the accuracy of muscle torques around the hip, knee and ankle joints predicted from these models were compared to those predicted from the same models containing generic data from elderly cadaveric specimens. (B) The root mean squared (RMS) errors of the outputs from the subject‐specific models were substantially lower than those from the generic models around all joints and through all movements tested, highlighting the importance of subject‐specific muscle architecture to reflect *in vivo* muscle functional capacity accurately (B).

However, whether scaled‐generic or subject‐specific, human or non‐human, how accurately these models reflect the dynamics of an individual is often dictated in part by the accuracy of the muscle architecture data that inform the dynamic behaviour of the MTUs within them. Indeed, various studies that have investigated the sensitivity of functional predictions from musculoskeletal measurements to changes in MTU force‐generating properties have shown that fibre length is a particularly crucial parameter to measure accurately (Curtis *et al*., 2010; Bates *et al*., 2010b; Bates & Falkingham, 2012; Groning *et al*., 2013; Charles *et al*., 2016b, 2020; Broyde *et al*., 2021). Indeed, notable improvements in certain model outputs (e.g. maximal muscle torques) have been demonstrated when using subject‐specific muscle architecture (fibre lengths, pennation angles, and PCSAs) data in musculoskeletal models (Charles *et al*., 2020) (Fig. 2). This study also highlighted the high sensitivity of musculoskeletal model outputs to their input values as well as, perhaps most significantly, the need to measure fibre lengths accurately to generate more reliable predictions of muscle functional performance.

#### (c) Comparative anatomy and zoology

Moving beyond studies of individual species, muscle architecture data has been used to make higher level inferences about adaptations and constraints in muscle anatomy and function across species (Fig. 1C). The approach of linking similarities and differences in muscle architecture to behavioural and ecological specialisations across species is common in studies of locomotion (Anapol & Gray, 2003; Crook *et al*., 2008; Hudson *et al*., 2011a,b; Allen *et al*., 2014; Paxton *et al*., 2014; Rosin & Nyakatura, 2017; Bohmer *et al*., 2018, 2019; Leischner *et al*., 2018; Bribiesca‐Contreras, Parslew & Sellers, 2019; Nyakatura *et al*., 2019) and mastication (Herrel *et al*., 2008; Taylor *et al*., 2009; Santana, Dumont & Davis, 2010; Becerra *et al*., 2011; Perry, Hartstone‐Rose & Wall, 2011; Hartstone‐Rose, Perry & Morrow, 2012; Ginot *et al*., 2018; Hartstone‐Rose *et al*., 2018; Meyers, Nishikawa & Herrel, 2018; Hartstone‐Rose, Hertzig & Dickinson, 2019), but has been less frequently pursued in the axial system [for examples see Webster, Hudson & Channon (2014) and Cuff *et al*. (2016a)].

In locomotor studies, analyses of muscular adaptations of species capable of high‐performance behaviours are particularly commonplace (Smith *et al*., 2006; Williams *et al*., 2007a, 2008a,b; Crook *et al*., 2008; Lamas *et al*., 2014), and provide insights into how feats such as exceptional running speeds are achieved by different animals. For example, Hudson *et al*. (2011a,b) explored the hypothesis that faster maximum running speeds in the cheetah (*Acinonyx jubatus*) *versus* the greyhound might be explained by differences in limb extensor muscle properties, including architectural parameters like fibre length, pennation angle, and PCSA. However, surprisingly, they found that while the hip extensor muscles of the cheetah have architectures and moment arms suitable for producing large joint torques, they were not systematically more powerful than in the greyhound. They suggest instead that the exceptional locomotor performance of the cheetah might be better explained anatomically by a range of skeletal features and functionally through greater power amplification generated through flexion–extension of the vertebral column (Hudson *et al*., 2011a,b).

However, ecomorphological studies of muscle architecture in limbs are not restricted to celebrated high‐performance animals. For example, a number of studies have examined the nature of functional modifications seen in limb muscles to facilitate flight (Bribiesca‐Contreras *et al*., 2019), arboreality (Anapol & Gray, 2003; Taverne *et al*., 2018) and fossoriality (Nyakatura *et al*., 2019). Allen *et al*. (2014) compared both fore‐ and hind limb muscle architecture in Crocodylidae and Alligatoridae to assess the role it may play in the use of asymmetrical gaits in the former group. Based on this comparison, they hypothesise that relatively longer muscle fascicles and smaller PCSAs may facilitate asymmetrical gaits in Crocodylidae by enabling large, rapid limb motions (Allen *et al*., 2014). Interestingly, these authors also note diverging ontogenetic trends in muscle architecture within these two groups. Ontogenetic changes in muscle architecture have also been studied in a range of other species [e.g. emu *Dromaius novaehollandiae* (Lamas *et al*., 2014); ostrich (Channon *et al*., 2019); eastern cottontail rabbit *Sylvilagus floridanus* (Butcher *et al*., 2019); rat *Rattus norvegicus domestica* (Woittiez *et al*., 1986)], including analyses of the contribution of differences in muscle properties to disparate gait, energetics and maximal performance in male *versus* female chickens across sexual maturity (Rose *et al*., 2016a,b).

Adaptive changes in muscle architecture have also been studied in a range of other selectively bred animals. Webster *et al*. (2014) recovered differences in the architecture of the epiaxial musculature of Staffordshire bull terriers and greyhounds, which appeared to be functionally adaptive to their selective breeding for physical combat *versus* high‐speed performance. Additionally, the impact of selective breeding practices in the meat industry on muscle architecture has been studied in chickens (Paxton *et al*., 2010, 2014), where a decline in relative maximal force‐generating capacity in meat‐bred chickens relative to junglefowl (*Gallus sonneratii*) has been reported and hypothesised to contribute significantly to reduced locomotor performance in these domesticated breeds (Paxton *et al*., 2010). Taylor, Vinyard & Payseur (2008) (see also Vinyard & Payseur, 2008) used selectively bred strains of mice to investigate plasticity and heritability in the architecture (fibre lengths, pennation angles, and PCSAs) of jaw‐closing muscles and correlated this with functional metrics such as maximum gape angle.

Comparative studies have also examined allometric patterns in muscle architecture, and the links between maximal force‐generating capacity and size‐related changes in locomotor performance. Alexander *et al*. (1981) measured limb muscle masses and fibre lengths (and other properties) in a large sample of mammals, ranging in body size from shrews to elephants. While finding some statistical support for isometric scaling in muscle properties, these authors highlighted potentially important adaptive differences in taxonomic and ecological groups with different locomotor repertoires (Alexander *et al*., 1981). By contrast, Maloiy *et al*. (1979) carried out a similar study of hind limb muscles in terrestrial birds and generally found allometric patterns consistent with elastic similarity, but noted wide confidence intervals in their regression analyses.

#### (d) Evolutionary biomechanics

The use of muscle architecture data in evolutionary and palaeontological studies of functional morphology and biomechanics fall broadly into one of two categories; comparative studies that use measured data from extant taxa to infer evolutionary changes in muscle properties and functions in extinct lineages; and biomechanical simulations of function and behaviours in fossil taxa.

**Fig. 3:**
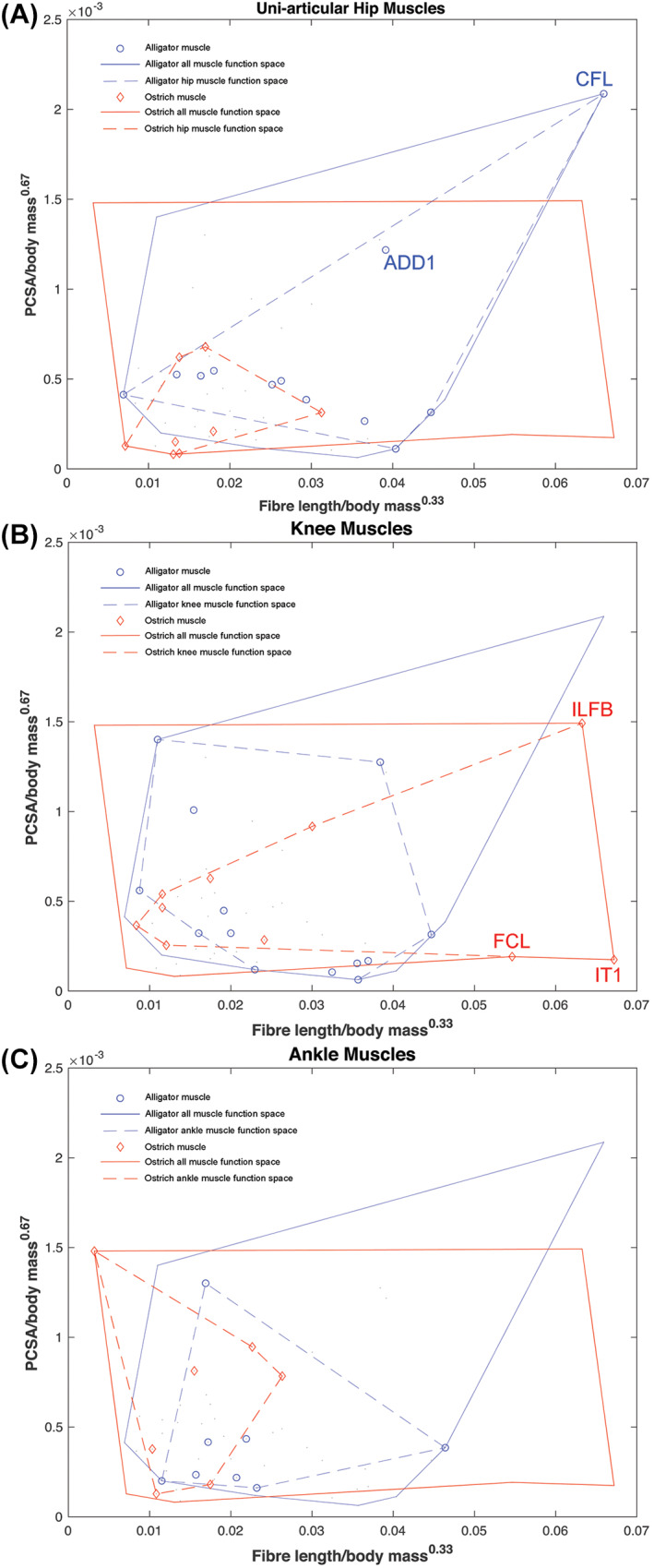
The concept of functional morphospace to examine muscle architectural functional specialisations. By relating the fibre length to the physiological cross‐sectional area (PCSA) of a muscle, it can be classified as either force specialised (short fibres, high PCSA), displacement specialised (long fibres, low PCSA), or power specialised (moderate to long fibres, moderate to high PCSA). Bates & Schachner (2012) found that the hip muscles of the alligator *Alligator mississippiensis* occupy a wider area of functional morphospace, and thus contain a wider range of architectural specialisations, than those in the ostrich *Struthio camelus*, (A). This pattern is reversed at the knee joint however, with the ostrich muscles displaying more adaptations for power and displacement specialisation than the alligator muscles (B). At the ankle, muscles are more force specialised in the ostrich compared to the more displacement specialised muscles of the alligator (C). Overall, these differences hint at possible adaptations of muscle architecture throughout evolutionary lineages. ADD, adductor femoris; CFL, caudofemoralis longus; FCL, flexor cruris lateralis; ILFB, iliofibularis; IT, iliotibialis.

The rarity of soft tissue preservation in the fossil record makes reconstructing the form and function of extinct vertebrates a challenging practice and largely restricts morphological analyses to the most durable skeletal materials (i.e. bone). The Extant Phylogenetic Bracket (EPB) approach was proposed by Witmer (1995) as a systematic means of inferring soft tissue features in extinct taxa and has been widely employed by researchers interested in understanding functional transitions documented in the fossil record. Stated explicitly, the development of soft tissue attributes in extinct taxa is judged by the presence of the same features in extant outgroups that phylogenetically bracket the fossil taxon of interest (Witmer, 1995). The EPB principle has also been used to gain insight into evolutionary changes in muscle architecture by quantitively comparing measured muscle properties in the first or most immediate outgroups of the fossil group of interest. For example, the differences in muscle architecture measured in representatives of extant crocodilians and birds have been used to constrain changes that might have occurred during the evolution of bipedalism in bird‐line dinosaurs (Bates & Schachner, 2012). This work used the muscle functional morphospace concept to examine specialisations in the architecture (fibre lengths and PCSAs specifically) of key muscles that might underpin the way extant groups habitually move (Fig. 3). The data highlights, among other things, that hip‐driven locomotion in the alligator *Alligator mississippiensis* is associated with hip muscles occupying a much larger area of total functional morphospace than those of the ostrich (Fig. 3A), which uses considerably less hip motion and powers the stride predominantly from the knee and ankle (Gatesy, 1990, 1991a,b). Particularly notable among hip muscles are the much greater specialisation for power (long fibres and large PCSAs) in the caudofemoralis longus (CFL) and adductor femoris muscles (particularly ADD1) in the alligator, which play prominent roles in propulsion and three‐dimensional limb control in the abducted limb postures used by crocodilians (Gatesy, 1991a; Hutchinson & Gatesy, 2000). This pattern of muscle specialisation is reversed at the knee joint, with the ostrich knee muscles occupying a greater area of functional morphospace and possessing a number of muscles with much higher levels of power and displacement (long fibres) specialisation than in the alligator (Fig. 3B). This is again consistent with the knee showing a greater range of motion and contribution to overall stride length during *in‐vivo* behaviours in birds compared to crocodilians (Gatesy, 1990, 1991a,b). Most ankle muscles show relatively weak specialisation in both the ostrich and alligator (Fig. 3C). However, the individual muscles and overall ankle muscle functional morphospace are noticeably more force specialised in the ostrich compared to the more general (and some instances extreme) displacement specialist architectures seen in the alligator (Fig. 3C). Greater force specialisation in the ostrich may be associated with digitigrade posture and an enhanced role of tendons in modulating MTU length change *in vivo*, such that ostriches (and other birds; Roberts *et al*., 1997) are able to generate greater stance‐phase mechanical power *via* the storage and release of elastic energy from the tendons of ankle muscles (Rubenson *et al*., 2011). Collectively, these relationships between muscle specialisation and joint contributions to gait provide qualitative insights into muscular adaptations that might have occurred during the evolution of bipedalism in dinosaurs (Hutchinson & Gatesy, 2000; Bates & Schachner, 2012; Allen, Kilbourne & Hutchinson, 2021). For example, basal theropod dinosaurs likely retained large (power specialised) tailed‐based femoral retractors similar to crocodilians, but as digitigrade bipeds may have already utilised more force‐specialised musculature and amplified elastic energy generation through tendons at the ankle (Bates & Schachner, 2012). Similar hypotheses regarding evolutionary changes in muscle mechanics in early mammals (Fahn‐Lai, Biewener & Pierce, 2020) and hominids (Payne *et al*., 2006; Strait *et al*., 2009; Myatt *et al*., 2011, 2012) have also been made based on comparisons of muscle architecture and function in EPB groups.

With the more widespread availability and affordability of digitisation approaches over the past two decades (Bates *et al*., 2010a; Falkingham, 2012), biomechanical modelling and simulation has become an increasingly popular means of quantitatively testing hypotheses about the functional capabilities of extinct animals, and how morphological change seen in the fossil record translates to changing mechanical performance. The major benefit of these models is the ability to derive absolute quantitative predictions of performance (e.g. bite force, running speed, metabolic costs of movement) by analysing all the major causative factors that underpin animal motion. As such, they require numerical values for anatomical and physiological factors that govern muscle force production, including muscle architecture (Fig. 1A, B). For this reason, palaeobiologists have used a variety of approaches and assumptions to derive quantitative values for muscle architecture (fibre lengths, pennation angles, and PCSAs) in biomechanical models of fossil taxa.

A popular approach, particularly in biomechanical evaluations of skull mechanics in extinct taxa, has been to reconstruct all muscles as parallel fibred with fibre lengths equal to the total length of the MTU at a specific length or joint posture (Button, Barrett & Rayfield, 2016; Gignac & Erickson, 2017; Adams *et al*., 2019; Chambi‐Trowell *et al*., 2020), and subsequently use these values in the calculation of PCSA (Fig. 1A). Other studies have also assumed uniform parallel‐fibre architecture across muscles but have used a variety of different data from extant animals to derive mean fibre length for their reconstructed muscles in extinct taxa. For example, in an analysis of limb biomechanics in fossil theropod dinosaurs, Hutchinson (2004b) set fibre lengths as the same proportion of the relevant segment length (thigh, shank etc.) measured in extant EPB taxa. Another approach, popular in studies of hominid evolution, is to scale fibre lengths (e.g. by body mass) directly from measured values in closely related extant taxa like humans or chimpanzees (Sellers *et al*., 2005; Strait *et al*., 2009, 2010; Crompton *et al*., 2012). Other studies have set fibre lengths to the proportion, or proportions, of total MTU measured in extant animals, and additionally examined the functional consequences of assuming parallel‐fibred *versus* pennate muscle architecture on hypothesis testing (Bates & Falkingham, 2012, 2018; Cost *et al*., 2020).

Conceptual or theoretical models of animal movement also require numerical values for muscle architecture, and given the generalised nature of these models (i.e. not specific to any particular real animal) researchers have often used functional principles based on the expected force–length characteristics of muscles (Fig. 1B) to derive values for fibre lengths. For example, Alexander (1995) used a generalised model to investigate the over‐arching mechanics of bipedal jumping and set resting fibre lengths as a proportion of the length change that each muscle would be expected to experience. Sellers *et al*. (2009) extended the same functional rationale to their reconstruction of limb muscles in the ornithischian dinosaur *Edmontosaurus*. Specifically, these authors set muscle fibre lengths to the value of MTU length change experienced when joints were rotated through their maximum flexion–extension ranges. They considered this a means of objectively generating fairly optimal fibre lengths for muscles since vertebrate muscles are typically able to generate force from approximately 60% to 160% of their resting length and would therefore be expected to work within this range *in vivo* (Fig. 1B). These authors later tested this assumption by examining the relationships between measured fibre length and total MTU length change in chimpanzees, greyhounds, ostrich and horses (Sellers *et al*., 2013). Overall, they found a modal value of between 0.4 and 0.6 for MTU extension/fibre length in these four species but noted that many muscles appeared to fall above and below this range (Sellers *et al*., 2013). This study was later extended by Bishop *et al*. (2021b) who carried out a similar assessment of the hind limb muscles of the tinamou and similarly suggested that simply setting fibre length as directly equal to MTU length change may not be strictly appropriate for most muscles.

The obvious implication of these different subjective choices about muscle architecture reconstruction is that longer fibre lengths and parallel‐fibred architecture will lead to lower maximal isometric forces (by yielding lower PCSAs; Fig. 1A) and different force–length and force–velocity profiles to muscles reconstructed with shorter fibre lengths and pennate architectures (Fig. 1B). However, despite the obvious impact on muscle force‐generating capacities, relatively few studies have examined how these subjective choices impact biomechanical predictions for extinct taxa (Bates *et al*., 2010b; Bates & Falkingham, 2012, 2018; Cost *et al*., 2020; Broyde *et al*., 2021). Where sensitivity analyses or direct comparisons of different approaches to architecture reconstruction have been carried out, they have tended to suggest the potential for relatively large uncertainty in predictions for parameters like muscle and bite force (Bates & Falkingham, 2012, 2018) and bone stress (Cost *et al*., 2020) within individual extinct species, which might limit the capacity of models to predict evolutionary patterns correctly (Broyde *et al*., 2021). The clear implication of these studies is a need to understand, through quantitative data, how muscle architecture is adaptively tuned in extant animals to better inform soft tissue and biomechanical reconstructions of fossil taxa (Bates & Falkingham, 2018; Broyde *et al*., 2021; Bishop *et al*., 2021b).

### (2) Methods of measuring muscle architecture

Muscle architecture, as defined by (Lieber & Fridén, 2000), can be gathered from individual muscles from a variety of species with two primary methods: traditional manual dissection of cadaveric specimens, or newer *in situ* or *in vivo* medical imaging‐based techniques, which each have distinct advantages and limitations.

#### (a) Manual dissection

Manual dissection has been the most common method to study anatomical form and function for over 1000 years, with pioneering anatomists such as Leonardo da Vinci using this approach as the foundation for detailed drawings of the human body. In more recent years, these dissection‐based approaches have been the basis of many seminal research papers detailing the architecture of human (Wickiewicz *et al*., 1983; Ward *et al*., 2009) and animal muscles (Sacks & Roy, 1982; Lieber & Blevins, 1989), as well as developing novel predictive models of how muscle fibres produce force (Hill, 1938; Zajac, 1989).

The process of measuring these muscle data from both human and animal cadaveric specimens can be broadly sub‐divided into two approaches: fresh *versus* fixed dissections. Fresh dissection typically involves removal of individual muscles from fresh/unfrozen specimens, followed by direct and immediate manual measurement of fibre properties by the investigator (e.g. using a ruler or callipers). Fixed dissection measurement typically involves a standardised set of procedures described by Sacks & Roy (1982) and Lieber, Fazeli & Botte (1990). Initially, the specimen is often immersed in a phosphate‐buffered formal saline solution to fix the muscle tissue. During this process, the joints can be locked at certain angles to ensure that any data measured from the muscles is specific to a physiologically appropriate pose. After fixation, the muscle or muscles of interest are carefully removed from the specimen and, if necessary, stored in phosphate‐buffered saline until measurement. Each muscle is then weighed to determine muscle mass (*M*_m_) and measured from origin to insertion to determine total muscle length (*L*_m_). Any external tendon is often removed prior to this step. To measure the lengths of individual muscle fascicles (bundles of 5–50 fibres), the muscles are then placed in a sulfuric acid solution partially to digest the connective tissue surrounding the muscle belly and fascicles. After digestion, the pennation angle (θ) of the muscle fibres can be measured from the surface of the muscle; multiple measurements are usually taken from different regions of the muscle (i.e proximal, middle, distal) to generate an average value. Individual muscle fascicles (considered to be functionally equivalent to individual muscle fibres) can then be isolated and measured to estimate muscle fibre length (*L*_f_). Similar to θ, these are taken from various regions of the muscle belly to generate an average value. For larger specimens (i.e. humans), these are usually measured using callipers, while for smaller animals the fascicles can be mounted on slides and measured under magnification (Burkholder *et al*., 1994; Charles *et al*., 2016a). For human muscles, it is then common to adjust these measured fibre length values to an optimal fibre length to account for any discrepancies between the measured fibre length and the length at which it produces its optimal force. This can be done by measuring the length of the sarcomeres within each extracted fibre bundle through laser diffraction (Lieber *et al*., 1990), which are then compared to an accepted optimal sarcomere length for skeletal muscle and used to generate L_f_ *’* for each muscle. All these data gathered for each muscle are then used to calculate their PCSA (Fig. 1A).

Arguably the principal limitation of these manual approaches is that gathering a large volume of muscle data can be time consuming and doing so in an accurate manner can require a high level of dissection skill. Furthermore, ensuring measurements, particularly of *L*_f_ and θ, are taken at either a resting or physiologically appropriate length is difficult (particularly if measuring from unfixed tissue), and there is a high probability of *post‐mortem* artefacts or damage to specimens if not stored correctly. For human muscle architecture data, the frequent use of cadavers biases these data towards being mostly obtained from elderly individuals, which somewhat reduces the applicability of the data to the wider population, particularly if the data are being used to infer muscle function in younger individuals.

#### (b) Imaging

These limitations of traditional dissection methods to measure muscle architecture, along with advances in technology, have led to the formulation of alternative frameworks to gather these data. These primarily involve using various medical imaging modalities such as ultrasound, MR or CT imaging to visualise muscles *in vivo* or *in situ*, which allows for the non‐destructive measurement of crucial muscle architectural properties.

Ultrasound imaging has been used extensively in humans to image the fibre arrangements *in vivo* within prominent muscle groups such as the quadriceps (Seymour *et al*., 2009; Noorkoiv, Nosaka & Blazevich, 2010) and triceps surae (Barber, Barrett & Lichtwark, 2009; Dick, Biewener & Wakeling, 2017), which have tested assumptions of the Hill‐type model of muscle force production (Dick *et al*., 2017) and investigated the tendency for pennate muscles to optimise θ to produce maximal force, known as ‘muscle gearing’ (Randhawa, Jackman & Wakeling, 2013). However, despite the low relative cost of ultrasound imaging, the small field of view and ability to only image the more superficial muscles of the musculoskeletal system limit its utility in fully characterising the muscle architecture within an individual.

Recent studies have shown that a combination of MRI sequences can be used to obtain detailed muscle force‐generating properties from every muscle within a large field of view in a valid and repeatable way and thus overcomes many of the limitations associated with ultrasound imaging (Bolsterlee, D'Souza & Herbert, 2019; Charles, Moon & Anderst, 2019a; Charles *et al*., 2020). The most important of these sequences is diffusion tensor imaging (DTI), which images different structures based on the relative diffusion of water through them and therefore allows for the visualisation and measurement of a large number of muscle fibres within a range of muscles (Froeling *et al*., 2012, 2015; Bolsterlee *et al*., 2015, 2018, 2019; Damon *et al*., 2016; Sieben *et al*., 2016; D'Souza *et al*., 2019; Charles *et al*., 2019a). When combined with an anatomical sequence to allow for the measurement of muscle volumes (e.g. T1 turbo spin echo), this MRI‐based framework can be used to gather extensive muscle architecture data from an individual *in vivo* (Charles, Suntaxi & Anderst, 2019b). However, while this framework has been validated for gathering muscle architecture data from healthy human muscle, it is unclear how effectively it could be used to gather similar data from pathological or dysfunctional tissue, which may contain damaged fibres or otherwise disrupt the diffusion of water, which would make the interpretation of such images somewhat unclear. Furthermore, while this methodology could theoretically be applied to other large animals, it is currently unclear how well the approach will scale down to smaller animals.

Instead, several studies have used iodine‐based contrast‐enhanced CT scanning (also known as diceCT; Gignac *et al*., 2016) to gather these data from smaller animals. This framework involves submerging a specimen in an iodine potassium‐iodide (I_2_KI) solution for an appropriate amount of time to optimise soft tissue contrast but minimise shrinkage artefacts (Vickerton, Jarvis & Jeffery, 2013) prior to CT or microCT scanning. This staining process enhances the contrast of soft tissue structures thus rendering individual muscles visible in these CT scans, from which various important anatomical variables (muscle volumes and fibre lengths) can be measured (Kupczik *et al*., 2015). This method has been used to quantify the muscle architecture and musculoskeletal geometry of, among others, the jaw musculature of the crab‐eating macaque (*Macaca fascicularis*) (Dickinson, Stark & Kupczik, 2018), the pectoral muscles of the European starling (*Sturnus vulgaris*) (Sullivan *et al*., 2019), various species of bat (Santana, 2018), as well as the hindlimbs of the mouse (Charles *et al*., 2016a) and the red‐legged running frog (*Phlyctimantis maculatus*) (Collings & Richards, 2019). Some of these frameworks (Dickinson *et al*., 2018; Sullivan *et al*., 2019) were able to produce estimates of muscle fibre lengths from imaging alone through a form of digital fibre tracking, which generated a sample size of fibre lengths equivalent to that produced from DTI. However, while diceCT has been successful for investigating the muscle architecture of many small species and specimens, it is limited to *in situ* staining and imaging of cadaveric tissue which has been shown to undergo a potentially substantial degree of shrinkage during the staining process (Vickerton *et al*., 2013), and the time needed to stain each tissue successfully also reduces its ease of applicability to larger specimens.

Overall, these medical imaging‐based methods of measuring muscle architecture hold several advantages compared to dissection methods, including the ability to automate imaging protocols to ensure repeatability both within and across studies within different specimens. The non‐destructive nature of these methods also eliminates the possibility of damaging important structures through human error, a common drawback of more manual methods. Additionally, particularly with regard to MR scanning, they are methods that can be used in the context of humans to measure muscle architecture from a wider range of demographics than is possible with dissection methods, including in young, healthy individuals (Charles *et al*., 2019b, 2020), and theoretically could be applied in more a comparative sense to rare or endangered animal species from which cadaveric specimens are difficult to obtain. However, it should be noted that these techniques often require the use of often prohibitively expensive equipment. For example, a 3T MRI scanner can cost around $3 million USD to purchase, with additional funds required for infrastructure and maintenance, and so are not as ubiquitously available as other methods. Furthermore, creating and optimising imaging protocols, if needed, can be time‐consuming and requires an expert in radiography or medical physics to achieve (particularly for MRI and CT), and is a crucial step in the process given how much the quality of the muscle data gathered from these methods is dependent on the quality of the initial images.

#### (c) Sample size

Ultimately, the method chosen to measure muscle architecture (e.g. dissection *versus* medical imaging) will be dictated by the goals of the study and equipment available, accounting (where possible) for the advantages and disadvantages of each method. However, certain experimental considerations apply universally to all methods, with one such important consideration being sample size. Throughout the literature reviewed above, studies have sought to quantify and compare mean values for muscle architectural parameters, such as *L*_f_ and θ (Fig. 1A, B) to make inferences of muscle function and adaptation within and across animals (Fig. 1C). For example, for fibre lengths, this involves measuring a number of fascicles within a muscle belly to generate a single representative value (e.g. a mean fibre length). The fundamental assumption, therefore, is that the sub‐sample of individual fibres measured is sufficient in number to characterise the central tendency of fibre lengths within that muscle accurately, and subsequently other gross properties like PCSA.

**Fig. 4:**
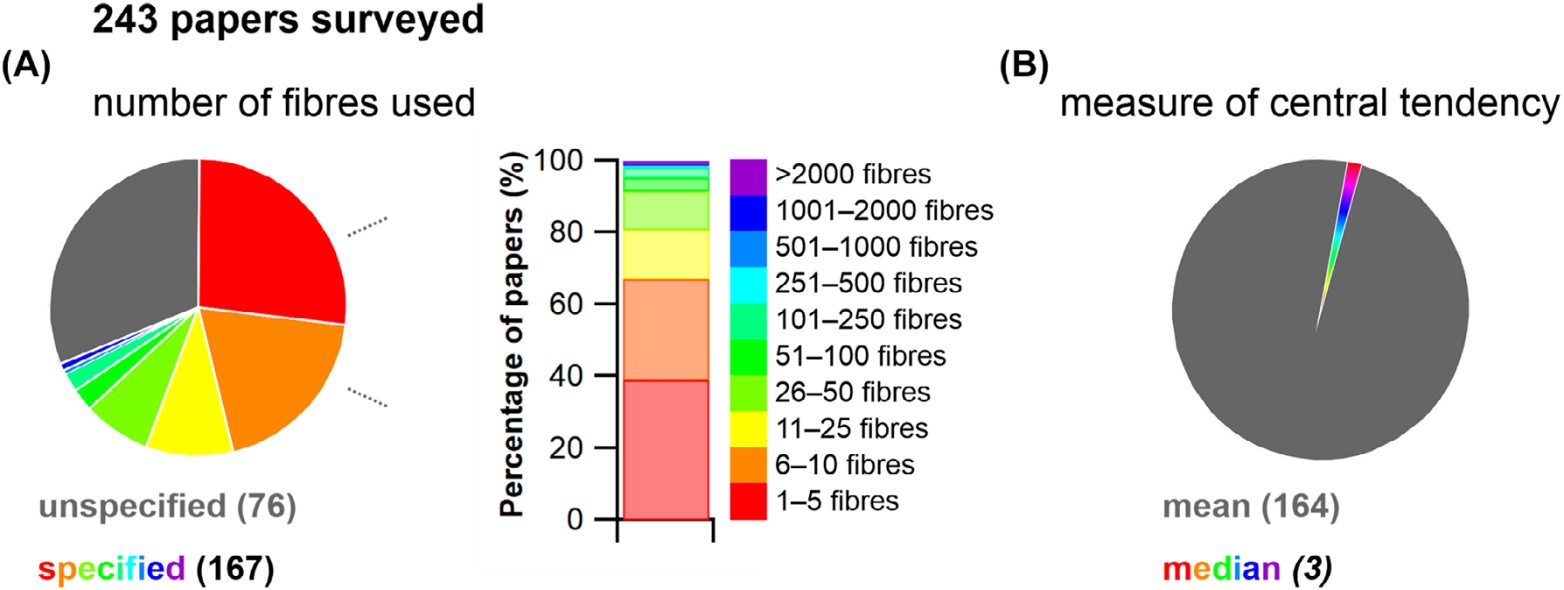
A review of the number of fibres per muscle measured to calculate mean architectural properties in 243 published studies. (A) From the studies that reported the number of muscle fibres used to calculate mean fibre length, 80.8% measured fewer than 25 fibres per muscle, with 38.9% measuring 5 fibres or fewer per muscle. Only 1.8% of the papers that reported their totals used over 250 fibres. (B) In addition, 99% of the 243 papers reported the mean fibre length, while only 1% reported the median.

Here, we surveyed 243 studies into vertebrate skeletal muscle (see online Supporting Information, Table S1) and found that the number of fibres used to generate mean values for architectural properties is usually extremely small (Fig. 4). We did not carry out a systematic review, rather we sought to sample as many papers as possible (prior to 2021) from across the research areas reviewed in the previous sections. Where studies reported the number of fibres measured, only 1.8% measured 250 fibres or more per muscle and only 17.4% used 25–250 measurements per muscle to generate a mean value. Thus the vast majority of studies (more than 80%) characterised muscle architectural properties based on less than 25 fibre measurements, with nearly 40% using 5 fibre measurements or fewer (Fig. 4A). In humans, it has been suggested that large muscles can contain up to ~400000 fibres (or ~600 fascicles) (Henriksson‐Larsen, Lexell & Sjostrom, 1983; Lexell, Downham & Sjostrom, 1986). Therefore, most studies have derived and analysed mean architectural properties for muscles based on a tiny fraction of the total number of fibres within those muscles (potentially <1% in some cases). It is also notable that of these 243 studies, only three calculated the median value of the initial fibre sample as a representation of a muscle's fibre length instead of the mean, despite the likelihood that fibre lengths are not normally distributed throughout a muscle (Young, Scott & Loeb, 1993; Schenk *et al*., 2013; Kupczik *et al*., 2015).

Breaking studies down by method reveals that both automated imaging and manual dissection‐based approaches have been used to measure relatively large numbers of fibres per muscle. For example, Rosin & Nyakatura (2017) measured up to 1754 fibres per muscle using manual dissection of fixed muscles. At least two other studies (Kim *et al*., 2007; Rosatelli, Ravichandiran & Agur, 2008) have taken more than 1000 measurements per muscle using the same manual approaches. On the other hand, it is surprising that approaches based on medical imaging data have not measured such high fibre numbers despite the potential for more rapid measurement using automated imaging tools, with Sullivan *et al*. (2019) and Dickinson *et al*. (2018) measuring a maximum of only 218 and 603 fibres, respectively. In fact, all methodological approaches for measuring muscle architecture have been used to measure only a small number of fibres, with zoological studies utilising fresh manual dissection almost always measuring fewer than 25 fibres per muscle (Table S1).

## III ASSESSING RELIABILITY ACROSS THE FIBRE TO FUNCTION PYRAMID OF INFERENCE

### (1) Framework and hypotheses

Measurements of muscle architecture are widely used across a range of biological disciplines to understand functional morphology at different scales. The near‐universal assumption that the mean value of a small proportion of fibre measurements can accurately represent the architectural properties of a muscle (Fig. 4; Table S1) has never been quantitatively tested. It therefore remains unknown whether potential inaccuracies in the representation of architectural properties derived from inadequate sample sizes impacts higher‐level interpretations of basic muscle function and how muscles are (or are not) adapted to power specific motions within and across species (Fig. 1C). In other words, it is presently unclear whether much of the biomechanics and muscle physiology literature over the past 80 years has made accurate interpretative leaps from fibre to function when analysing skeletal muscle.

**Fig. 5:**
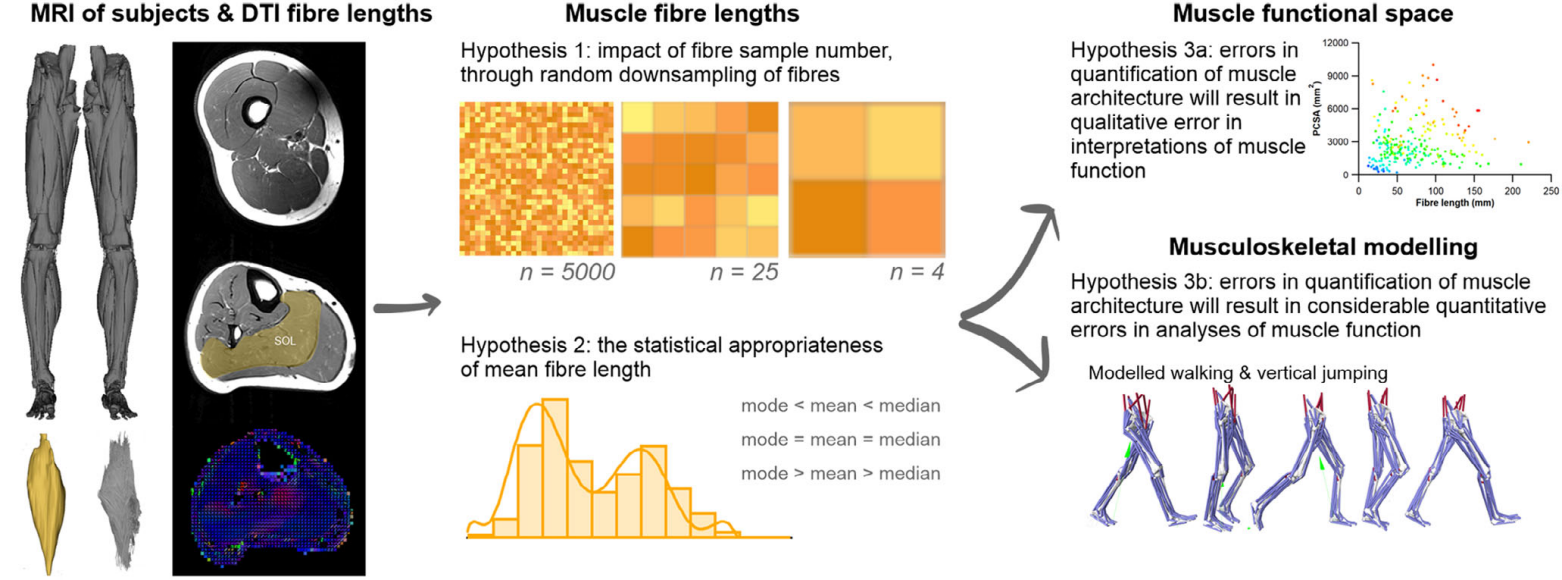
Overview of our experimental approach. T1 magnetic resonance imaging (MRI) was used to generate three‐dimensional meshes of 25 muscles of the lower limb of 10 subjects, and diffusion tensor imaging (DTI) was used to generate a sample of up to 5000 fibres from each of these muscles. To address Hypothesis 1, 1000 random subsamples of 5, 10, 50, 100, 250, 500, 1000 and 2000 fibres from the full sample were taken to assess the effect of fibre sample size on calculations of mean muscle fibre length and interpretations of functional specialisations. To address Hypothesis 2, the distributions of fibres within each muscle were assessed to quantify the statistical appropriateness of calculating mean or median fibre lengths to generate a single representative value. The functional implications of these two major assumptions often made during the collection of muscle architecture data, were tested using muscle function plots (Hypothesis 3a) to compare human and chimpanzee muscles, and musculoskeletal modelling and simulations (Hypothesis 3b), where muscle fibre dynamics during walking and jumping movements were predicted, and the potential errors introduced by these assumptions on these output metrics were calculated. PCSA, physiological cross‐sectional area; SOL, soleus.

To address these fundamental issues, we apply a novel combination of state‐of‐the‐art medical imaging, image and statistical analysis, and physics‐based computer simulation of human walking and jumping (Fig. 5) to quantify the potential errors in fibre length measurements, and the potential impacts of these errors on muscle functional predictions. First, we used DTI and deterministic fibre tractography (Bolsterlee *et al*., 2019; Charles *et al*., 2019a) to derive the architectural properties of a sample of human lower limb muscles using a large number of fibre measurements per muscle [approximately two and half times more than the highest used in previous studies (Rosin & Nyakatura, 2017)]. Second, using statistical analysis we determined the central tendency and distribution of fibre lengths and examined its vulnerability to sample size using subsampling simulations. Finally, we quantified the implications of inaccuracies in fibre architectures due to low sample sizes using a combination of dynamic simulations of human walking and jumping (to assess within‐species effects) and muscle function plots (to assess across‐species effects). We applied these approaches to test three hypotheses. First (HYP1), we hypothesise that measuring a small number of fibres (e.g. <25) per muscle may result in a substantial error in quantification of the central tendency of fibre architecture (i.e. an inaccurate mean value). Second (HYP2), based on the irregular and complex design of many muscles, as well as some previous muscle architecture studies (Young *et al*., 1993; Schenk *et al*., 2013; Kupczik *et al*., 2015), we theorise that the central tendency of muscle architectural properties is mostly, and perhaps always, more appropriately represented by the median value, rather than the mean, which has been used in 99% of studies surveyed here (Fig. 4B; Table S1). Finally, our third hypothesis proposes that the errors in the quantification of muscle architecture identified in HYP1 and HYP2 will result in considerable qualitative (HYP3a) and quantitative (HYP3b) error in higher‐level interpretations of muscle functional capacity, fibre dynamics and overall interpretations of muscle specialisation (Fig. 4C).

### (2) Methods

Data were gathered from 10 human subjects [Table S2; 5 male, 5 female; mean ± SD age 29 ± 3.7 years; body mass 67.9 ± 9 kg; height 175 ± 7 cm; body mass index (BMI) 21.9 ± 1.6 kgm^−2^] who gave informed consent prior to participating in the study in accordance with ethical approval from the University of Liverpool's Central University Research Ethics Committee for Physical Interventions (Reference number: 3757). A previously validated framework (Charles *et al*., 2020) was used to estimate subject‐specific muscle architecture data from 25 muscles of the right lower limb from each subject (Fig. 5A). This involved the use of two MRI sequences: T1‐weighted anatomical turbo spin‐echo (TSE) to estimate muscle volumes and visualise muscle attachment points, and DTI to estimate muscle *L*_f_, θ and PCSA (see Appendix S1 for sequence parameters and details regarding image analysis and processing). The validity and accuracy of this and similar 3D techniques has been established previously (Kupczik *et al*., 2015; Bolsterlee *et al*., 2019; Charles *et al*., 2019a, 2020), and it carries the advantage of allowing the *in vivo* measurement of a large number of individual fibres (>3000 per muscle). The general framework is described in detail in Appendix S1 and in Charles *et al*. (2020).

#### (a) Muscle fibre subsampling

**Fig. 6:**
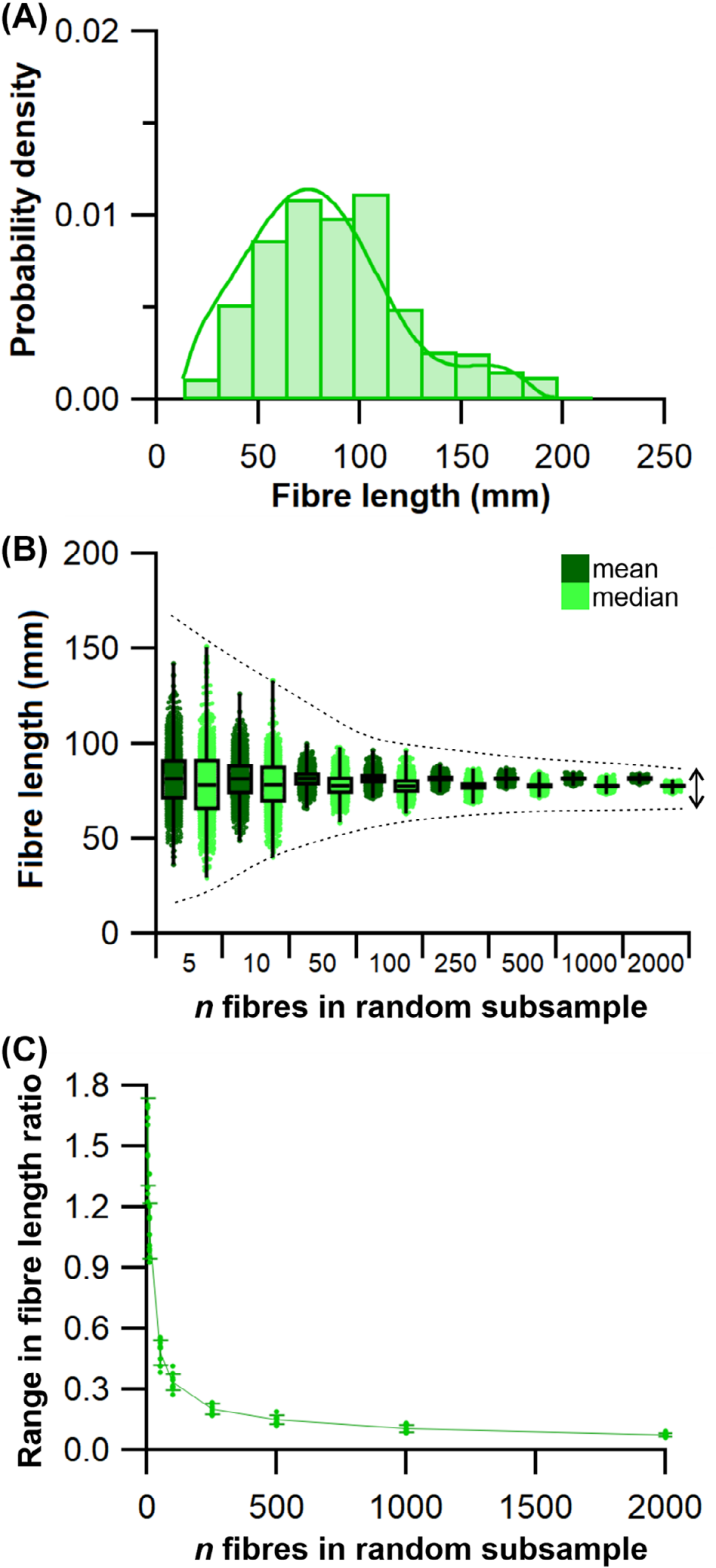
An example of the approach used to quantify potential errors in fibre length measurements due to sample size. (A) Example distribution of fibre lengths within a single muscle. The distributions of fibre lengths within each muscle were studied to assess the appropriateness of calculating mean or median of these measured fibres and address Hypothesis 2 (HYP2). (B, C) Random subsamples of the full set of muscle fibres generated from diffusion tensor imaging were used to study the possible range of mean and median fibre lengths obtainable from different initial sample sizes, expressed as a fraction of the ‘true’ mean or median value, to address Hypothesis 1 (HYP1).

To assess the range of both mean and median fibre length values possible from measuring only a subsample of muscle fibres (HYP1), 1000 random subsamples of 5, 10, 50, 100, 250, 500, 1000 and 2000 fibres from the full initial sample were obtained for each lower limb muscle from each subject (Figs 5B, 6B, C). The ranges of possible mean and median fibre length values obtained from each random subsample were calculated for each subject (Fig. 6B). The ‘accuracy’ of these values, here reported as these values relative to the ‘true’ mean and median value (from the full sample of fibres) and expressed as a ratio, were also calculated (Fig. 6C).

#### (b) Means versus medians

To test whether fibre lengths were normally distributed within a muscle belly (HYP2), Shapiro–Wilk tests were performed on the post‐processed fibre lengths to assess the distribution of fibre lengths within each muscle of the lower limb (e.g. Fig. 6A). Here, *P* ≤ 0.05 indicates that the distribution of fibres within a muscle is significantly different from a normal distribution and would suggest that mean values are not appropriate to represent the fibres within a particular muscle. Statistical tests were carried out in SPSS software (IBM Corp. IBM SPSS Statistics for Windows, Version 25.0).

#### (c) Climbing the pyramid of inference: from fibre to function

To examine the impact of results from HYP1 and HYP2 on quantitative predictions of muscle kinetics and energetics (HYP3b) within humans we used subject‐specific musculoskeletal modelling within the Opensim 4.1 (Seth *et al*., 2018) framework (Fig. 5). A previously constructed 92 MTU actuated personalised lower limb musculoskeletal model (Charles *et al*., 2020) (male, age 35 years, body mass 68 kg, height 176 cm, BMI 21.95 kgm^−2^) was used to predict muscle fibre lengths, forces and velocities throughout one stride of level walking (self‐selected speed of 1.4 m s^−1^) and one vertical jump (height 39 cm) using static optimisation. In this model, *L*_f_ and θ for each muscle actuator were defined by mean values from the fibres tracked from DTI (Model^mean^), with the *L*_f_ values then normalised to sarcomere length to generate an optimal fibre length [see Charles *et al*. (2020) for further details regarding normalisation). The muscle actuator force‐generating properties within this musculoskeletal model were then edited to create three additional model conditions, where actuator properties were informed by: (*i*) the maximum possible mean fibre length values from the subsamples of 5 fibres [*L*_f_^max5^; Model^max5^ (HYP1)], (*ii*) the minimum possible mean fibre length values from the subsamples of 5 fibres [*L*_f_^min5^, Model^min5^ (HYP1)] and (*iii*) the median fibre length values from the full sample of muscle fibres [*L*_f_^median^, Model^median^ (HYP2)]. For the Model^max5^ and Model^min5^ conditions, maximum isometric force values [*F*_max_, which is directly proportional to PCSA; see Charles *et al*. (2020) for more details] were recalculated based on these new *L*_f_ values in five muscles of the lower limb [gluteus maximus (Gmax); adductor magnus (AM); vastus lateralis (VL); tibialis anterior (TA); and soleus (SOL)], and tendon slack length (*L*_ts_) values for each muscle were recalculated using the same optimisation algorithm (Manal & Buchanan, 2004). In the Model^median^ condition, these properties were changed for all lower limb MTUs. Kinematic and kinetic data for walking and jumping performed by the human subject were collected using a 12‐camera motion capture system (Qualisys Inc.) and embedded force plates (Kistler).

The changes in functional outputs of each MTU in each model condition were quantified in two ways. Firstly, the root mean squared errors (RMSE) of the predicted MTU contractile dynamics (fibre force and normalised fibre length) were calculated during walking and jumping for Model^mean^, Model^max5^ and Model^min5^ relative to Model^median^,

(1) $$\text{RMSE}=\sqrt{\bar{{\left({\text{Model}}^{\text{median}}-{\text{Model}}^{x}\right)}^{2}}},$$

where Model^*x*^ refers to outputs from either Model^mean^, Model^max5^ or Model^min5^. As it was hypothesised that median fibre length would be more appropriate than means (HYP2) and this hypothesis was supported (see Section IV.2), the outputs from Model^median^ were assumed to be the ‘gold standard’ outputs against which errors were calculated from the other model conditions.

Secondly, from the positive and negative mechanical work generated by the fibres of each MTU in each model condition, their functional roles were quantified through the calculation of four dimensionless functional indices: strut, spring, motor and brake (Qiao & Jindrich, 2016; Lai, Biewener & Wakeling, 2019). Calculating these functional indices and the relative mechanical work generated by a muscle provides an approximation of their contribution to the flow of mechanical energy through the musculoskeletal system during dynamic movements. Comparing these values provides an overview of the influence of fibre length on inferred muscle function and changes in contribution to walking and jumping movements (see Appendix S1 for more details). These indices were calculated through both walking and jumping for each MTU with altered force‐generating properties (*L*_f_^max5^, PCSA^max5^, *L*_f_^min5^, PCSA^min5^) as well as each MTU with unaltered properties (*L*_f_^mean^, PCSA^mean^), in order to observe the effect that uncertainties in the measurement of the muscle architecture of a particular MTU have on the predicted functional capacity of another MTU.

We supplemented these within‐species (human) analyses with simple calculations of muscle function specialisation (as reviewed in Fig. 3), and this analysis was also extended to examine the impact of sample size (*n* fibres) on comparative (across species) inferences of muscle function, thereby assessing the implications of findings from HYP1 and HYP2 on the highest levels of the fibre to function pyramid of inference (Fig. 1C). First, we recalculated the PCSA of the Gmax, AM, VL, TA and SOL muscles in subject 1 using both the maximum mean fibre length values from subsamples of 5 fibres (*L*_f_^max5^, PCSA^max5^) and the minimum mean fibre length values from subsamples of 5 fibres (*L*_f_^max5^, PCSA^min5^). From the relationship between *L*_f_ and PCSA and the *L*_f_:PCSA ratio, it was possible to infer the functional specialisations of these five muscles and how this changed depending on initial *L*_f_ sample size using muscle function plots, similar to previous studies (Wickiewicz *et al*., 1983). Here, muscles with long *L*_f_ and low PCSA (high *L*_f_:PCSA ratio) were classed as displacement specialised, short *L*_f_ and high PCSA (low *L*_f_:PCSA ratio) as force specialised and long *L*_f_ and high PCSA as power specialised. To place these values in a comparative context, these relationships were compared to those from a common chimpanzee (*Pan troglodytes*) (O'Neill *et al*., 2013). This provided a between‐species comparison of the functional specialisations of lower limb muscles and allowed the exploration of potential errors in these comparisons that could be introduced by measuring only a small subset of a muscle's fibres.

## IV RESULTS AND DISCUSSION

Tables S3–S12 provide data on the fibre architecture and distributions for each of the study participants. Table S13 shows these data averaged across all subjects. Table S14 provides all‐subject averages and percentage differences in fibre length and PCSA between the mean and median values of the full sample of fibres, and the maximum and minimum possible mean and median values from random subsamples of 5 fibres.

### (1) How does fibre sample size influence mean architectural properties?

**Fig. 7:**
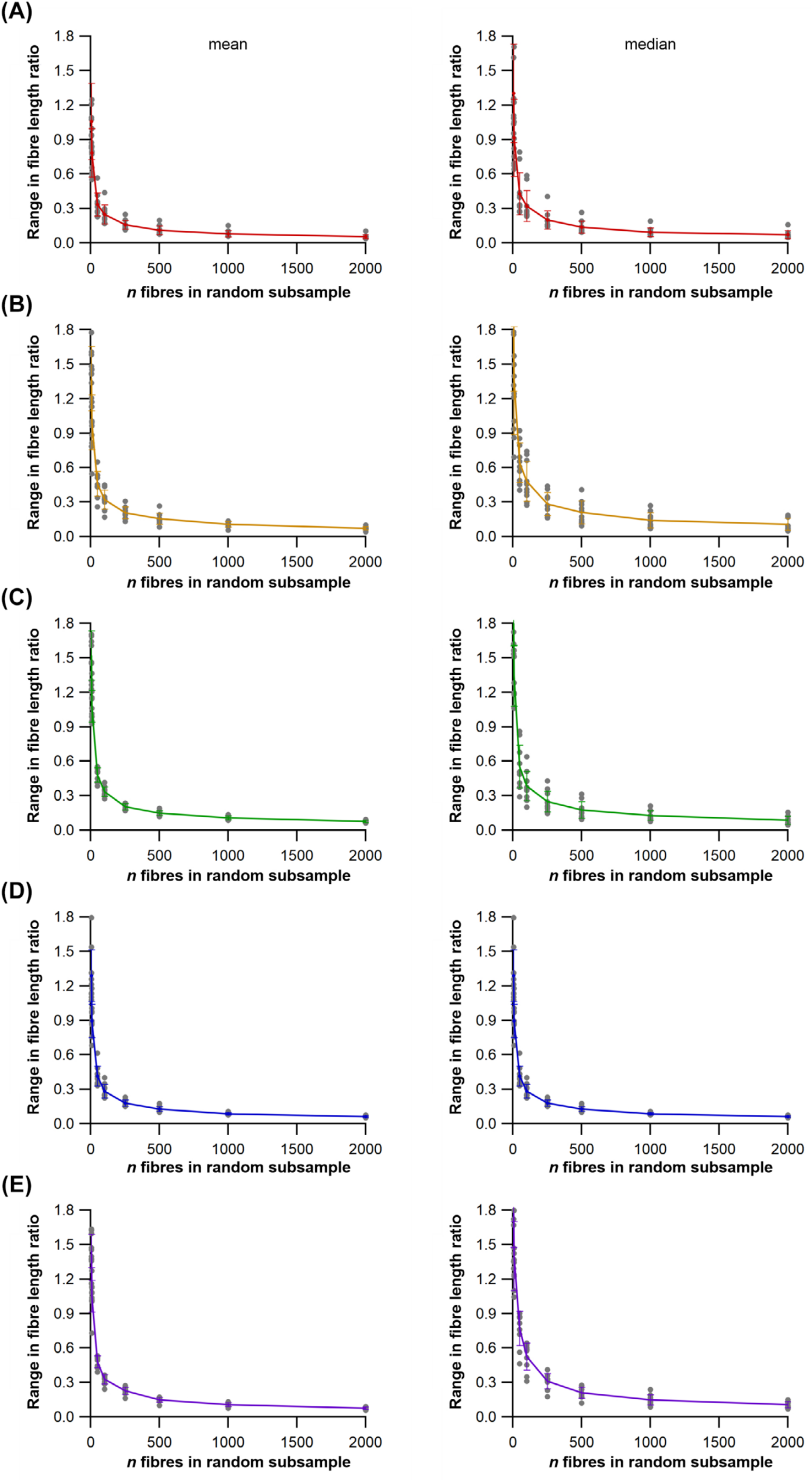
The effect of fibre number (*n* fibres) on mean and median fibre length. The effects on mean and median fibre length calculations from measuring random subsamples of 5, 10, 50, 100, 250, 500, 1000 and 2000 fibres from the full sample of fibres from the gluteus maximus (Gmax; A), adductor magnus (AM; B), vastus lateralis (VL; C), tibialis anterior (TA; D) and soleus (SOL; E) from all 10 subjects (lines represent data set means). For all muscles, the range of mean or median fibre lengths, expressed as a fraction of the ‘true’ mean or median value, was high in random subsamples of 5 fibres, but substantially lower in subsamples of >500 fibres.

This analysis shows that the range in both mean and median fibre lengths within the 1000 randomly generated subsamples remains narrowly distributed around the values given by the full sample of fibres in all muscles tested when *n* fibres is above 1000 per muscle (Figs 6, 7; Tables S15–S25). At *n* fibres <250 the range in mean and median fibre lengths increased considerably in an approximately exponential fashion, indicating that the values used to represent the fibre length of a muscle are highly sensitive to the particular fibres included within any measured sample (Figs 6, 7; Tables S15–S25). These patterns were consistent across all muscles of the lower limb and in all subjects, with a wide range of errors (expressed as percentage error) potentially attainable by measuring only a small sample of fibres from all muscles tested. For instance, the maximum mean or median fibre length values from samples of 5 fibres (*L*_f_^max5^) across all muscles in all subjects were on average 82% and 66% longer than the respective value from the full sample (Table S14), while taking the minimum possible mean fibre length value from these small subsamples (*L*_f_^min5^) led to fibre lengths that were on average 63% and 74% shorter than the original mean or median value. This degree of error varied between muscles however, with the more distal muscles of the limb showing higher potential errors (e.g. ankle dorsiflexors; mean = 123%/−57%; median = 155%/−69%) than the more proximal groups (e.g. knee extensors; mean = 57%/−69%; median = 94%/−76%). On an individual muscle level, the largest potential errors in attainable fibre length values were within the flexor digitorum longus (FDL), which ranged from −64 to 128% of *L*_f_^mean^ and −68 and 203% of *L*_f_^median^. The smallest errors were in the Gmax, which ranged from −62 to 33% of *L*_f_^mean^ and −75 to 40% of *L*_f_^median^ (Table S14).

These potential errors in estimates of fibre length translated to substantial differences in PCSA (PCSA^max5^, PCSA^min5^). The largest errors from PCSA^max5^ relative to PCSA^mean^ and PCSA^median^ were both in the EDL (mean = −60%; median = −66%), while the largest errors from PCSA^min5^ values were seen in the SOL (274%) and biceps femoris – long head (BFL; 628%) relative to PCSA^mean^ and PCSA^median^ values respectively. For the absolute ranges in these potential errors in both *L*_f_ and PCSA for each muscle, see Tables S3–S14.

**Fig. 8:**
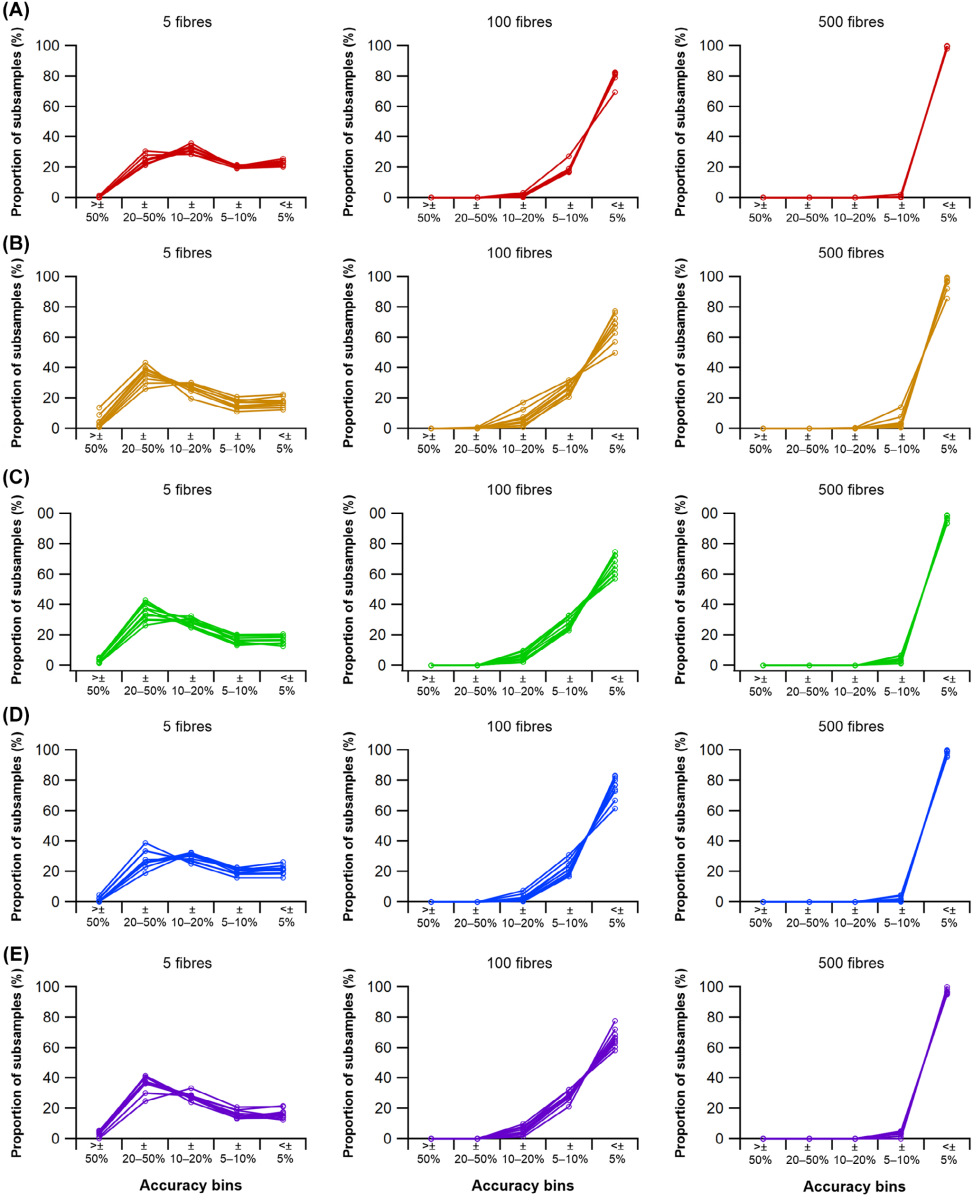
The influence of fibre sample numbers on mean fibre length across 10 subjects. The percentage of mean fibre length values within random subsamples of 5, 100 and 500 fibres for the gluteus maximums (A), adductor magnus (B), vastus lateralis (C), tibialis anterior (D) and soleus (E) muscles relative to the mean value from the full set of fibres, i.e. falling within ‘accuracy bins’ indicating how close the randomly subsampled values were to the ‘true’ mean.

To quantify the probability by which different *n* fibres sample sizes generated certain levels of error in mean fibre lengths we calculated the frequency with which error magnitudes across the 1000 randomly generated samples fell within a range of ‘error bins’ at each *n* fibres size (Fig. 8). Here, the probability that the mean fibre length value obtained from a sample of measured fibres is within an ‘error bin’ closer to the mean value from the full sample of fibres increases substantially with increasing sample size. For instance, for the 1000 subsamples of 5 fibres, 34.1% of the possible mean values fell within 20–50% of the ‘true’ mean when averaged across all muscles within all subjects. However this probability fell to 0.2% with samples of 100 fibres, and was 0% for samples ≥500 fibres (Fig. 8; see Tables S15–S24 for these data from individual subjects, and Table S25 for all‐subject and all‐muscle averages). Conversely, the probability of generating a fibre length value within 5% of the full mean was 100% with samples of 2000 fibres for all muscles, although this fell gradually with sample size to a probability of 17.4% on average over all muscles with samples of 5 fibres. The highest probability of obtaining a mean value within 5% of the true mean from these small samples was in the Gmax (22.9%), while the lowest probability was in the tibialis posterior (TP; 14.1%).

Collectively, these data provide strong support for the hypothesis (HYP1) that using small samples of muscle fibres may not accurately characterise the architecture of a muscle. Using samples as small as 5 fibres to generate either a mean or median fibre length value for the entire muscle decreases the probability of obtaining a value close to the ‘true’ value considerably and also potentially introduces degrees of error high enough to change estimations of a muscle's PCSA substantially. Only in subsamples of >500 fibres were ~100% of the mean fibre length values within ±5% of this ‘true’ mean. This number is more than 20× the *n* fibres that are usually measured in muscle architecture studies (Fig. 4A, Table S1). Our data also suggest that even measuring 100 fibres per muscle (e.g. Baker & Hall‐Craggs, 1978; Gollnick *et al*., 1981; Hermanson & Hurley, 1990), i.e. ~5–10 times more than the number measured in most studies with a few exceptions, may not be sufficient to obtain a representative mean or median fibre length value.

### (2) Are the central tendencies of muscle architectural properties typically more appropriately represented by the median or mean value?

**Fig. 9:**
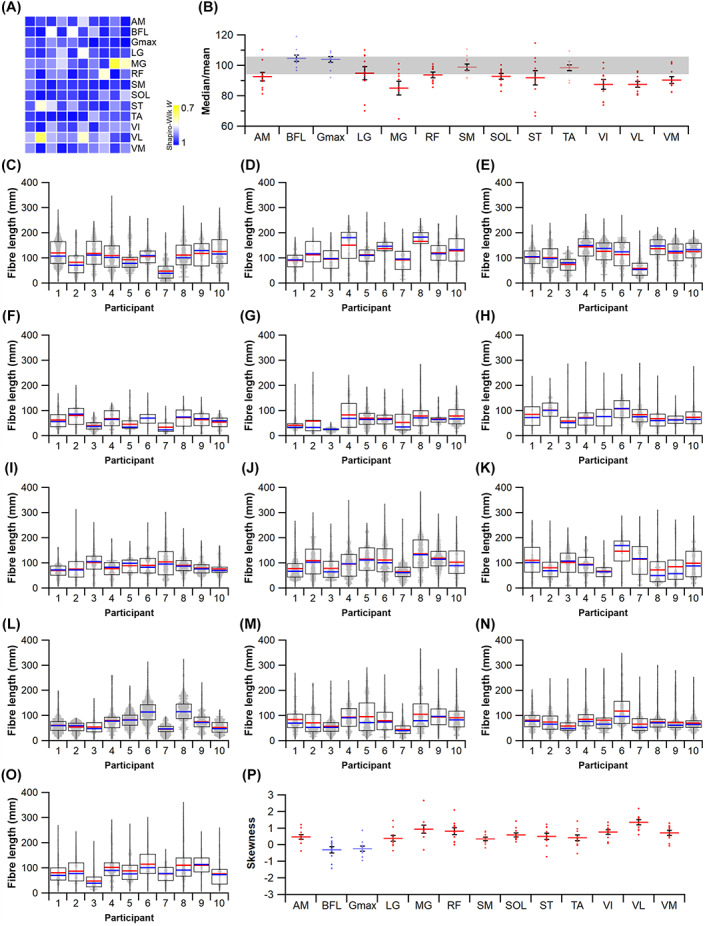
Statistical appropriateness of the use of mean fibre length. (A) Every muscle tested in this study violated the Shapiro–Wilk test for normality, indicating that no distribution of sampled fibres was normally distributed, despite a wide range of W statistics for these samples. (B) Dividing the median fibre length by the mean highlights the gross overestimation of fibre lengths when the mean is used, with data points falling outside the grey bar indicating ±5% difference from the mean. (C–O) Distributions of fibre lengths for all 10 participants across the adductor magnus (C), biceps femoris (long head) (D), gluteus maximus (E), lateral gastrocnemius (F), medial gastrocnemius (G) rectus femoris (H), semimembranosus (I), soleus (J), semitendinosus (K), tibialis anterior (L), vastus intermedius (M), vastus lateralis (N) and vastus medialis (O). Mean fibre length (red horizontal line) and median fibre length (blue horizontal line) are presented for each boxplot. (P) Skewness for each individual across each muscle. AM, adductor magnus; BFL, biceps femoris (long head); Gmax, gluteus maximus; LG, lateral gastrocnemius; MG, medial gastrocnemius; RF, rectus femoris; SM, semimembranosus; ST, semitendinosus; SOL, soleus; TA, tibialis anterior; VI, vastus intermedius; VL, vastus lateralis; VM, vastus medialis.

Shapiro–Wilk tests were carried out on the fibre length distributions within 25 muscles from each of the 10 subjects to test for normality. The results indicate that none of these 250 muscles showed a normal distribution of fibre lengths (Fig. 9; Tables S3–S12). Therefore, using medians to obtain a single representative value of a muscle's fibre length has more statistical support than using a mean value. Across all muscles in all 10 subjects, median fibre lengths were on average 4 mm shorter than mean fibre lengths, which translates on average to a 10% increase in PCSA (266 mm^2^) when using median fibre lengths to calculate muscle force‐generating properties relative to using mean values (Tables S3–S13). This discrepancy was variable between muscle groups, with an average difference of −10 mm/541 mm^2^–10%/13%) in the knee extensors, but only <1 mm/29 mm^2^ (−1%/3%) in the hip extensors (Table S14). In individual muscles, the largest average error was seen in the iliacus muscles, where a 9% error in *L*_f_ (4 mm) by measuring the mean value resulted in a 31% lower PCSA (916 mm^2^) relative to median values (Table S14). Collectively, this represents strong statistical support for the hypothesis (HYP2) and that the central tendency of muscle architectural properties is more appropriately represented by the median value rather than the mean, although the potential effects on fibre lengths and PCSA are much less than those of sample size.

### (3) How do fibre sample size‐related errors impact upon higher‐level interpretations of muscle function within a species?

**Fig. 10:**
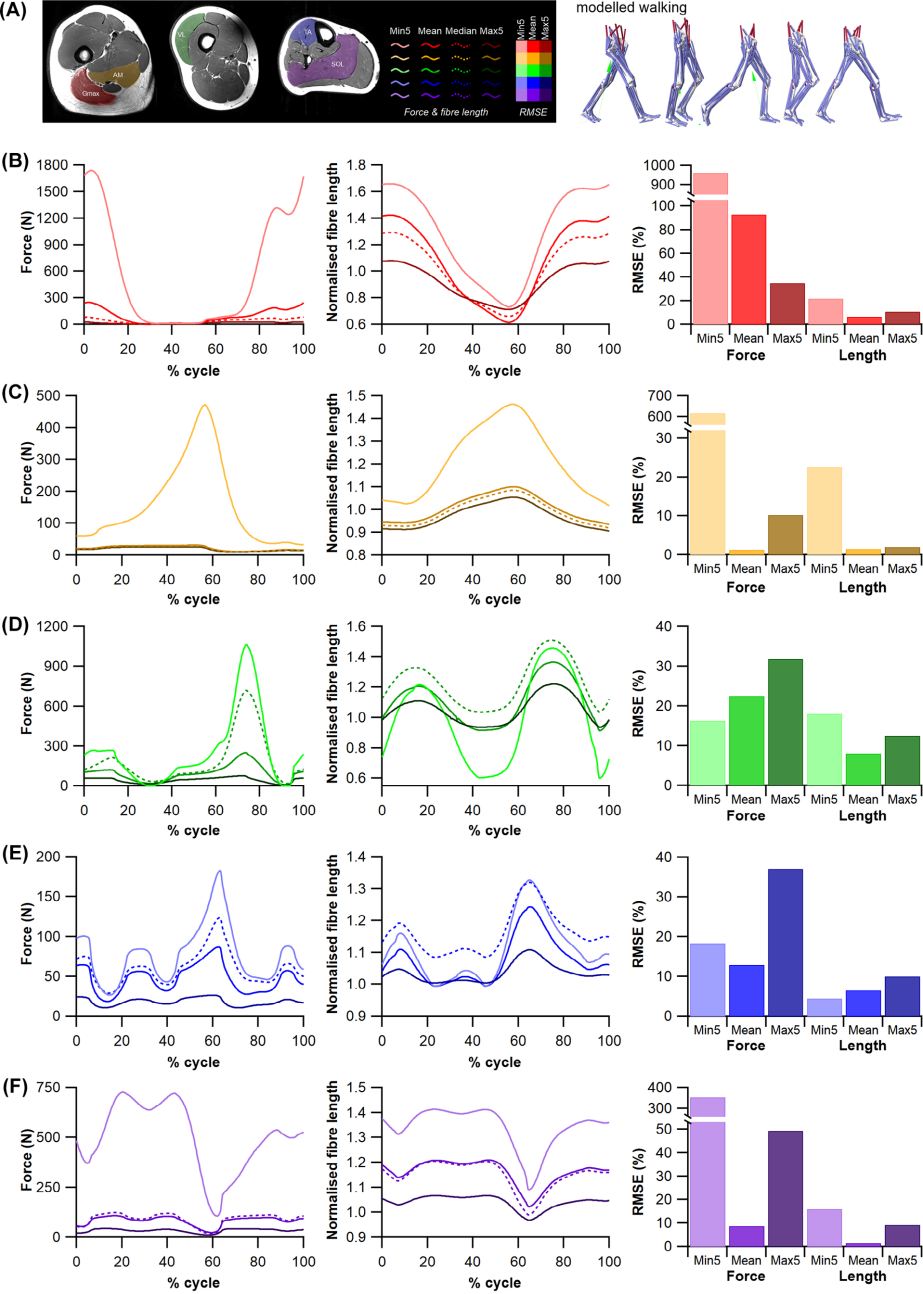
The influence of inaccurate fibre length measurements on simulations of walking. (A) Musculoskeletal modelling for walking. (B–F) Muscle fibre lengths and forces of the gluteus maximus (mid portion) (B), adductor magnus (hamstring part) (C), vastus lateralis (D), tibialis anterior (E) and soleus (F) predicted by static optimisation simulations of walking in models informed by the full mean value (Model^mean^, mid‐tone line), median value (Model^median^; mid‐tone dashed line), and the maximum (Model^max5^; darkest line) and minimum (Model^min5^; palest line) possible values from random subsamples of 5 fibres. The root mean squared errors (RMSEs) of these variables relative to Model^median^ are shown expressed as a percentage of the maximum Model^median^ value.

**Fig. 11:**
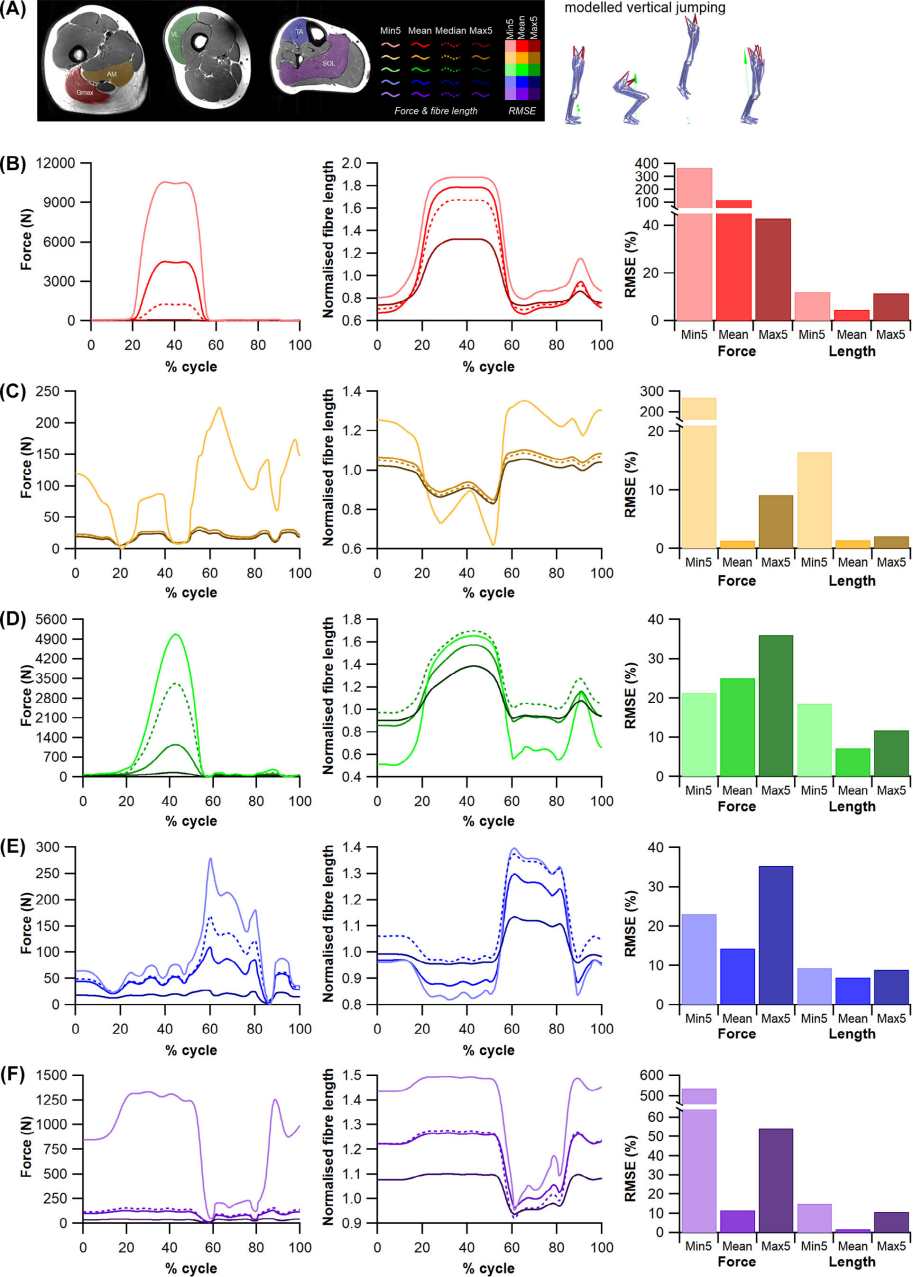
The influence of inaccurate fibre length measurements on simulations of vertical jumping. (A) Musculoskeletal modelling for vertical jumping. (B–F) Muscle fibre lengths and forces of the gluteus maximus (mid portion) (B), adductor magnus (hamstring part) (C), vastus lateralis (D), tibialis anterior (E) and soleus (F) predicted by static optimisation simulations of jumping in models informed by the full mean value (Model^mean^, mid‐tone line), median value (Model^median^; mid‐tone dashed line), and the maximum (Model^max5^; darkest line) and minimum (Model^min5^; palest line) possible values from random subsamples of 5 fibres. The root mean squared errors (RMSEs) of these variables relative to Model_median_ are shown expressed as a percentage of the maximum Model_median_ value.

**Fig. 12:**
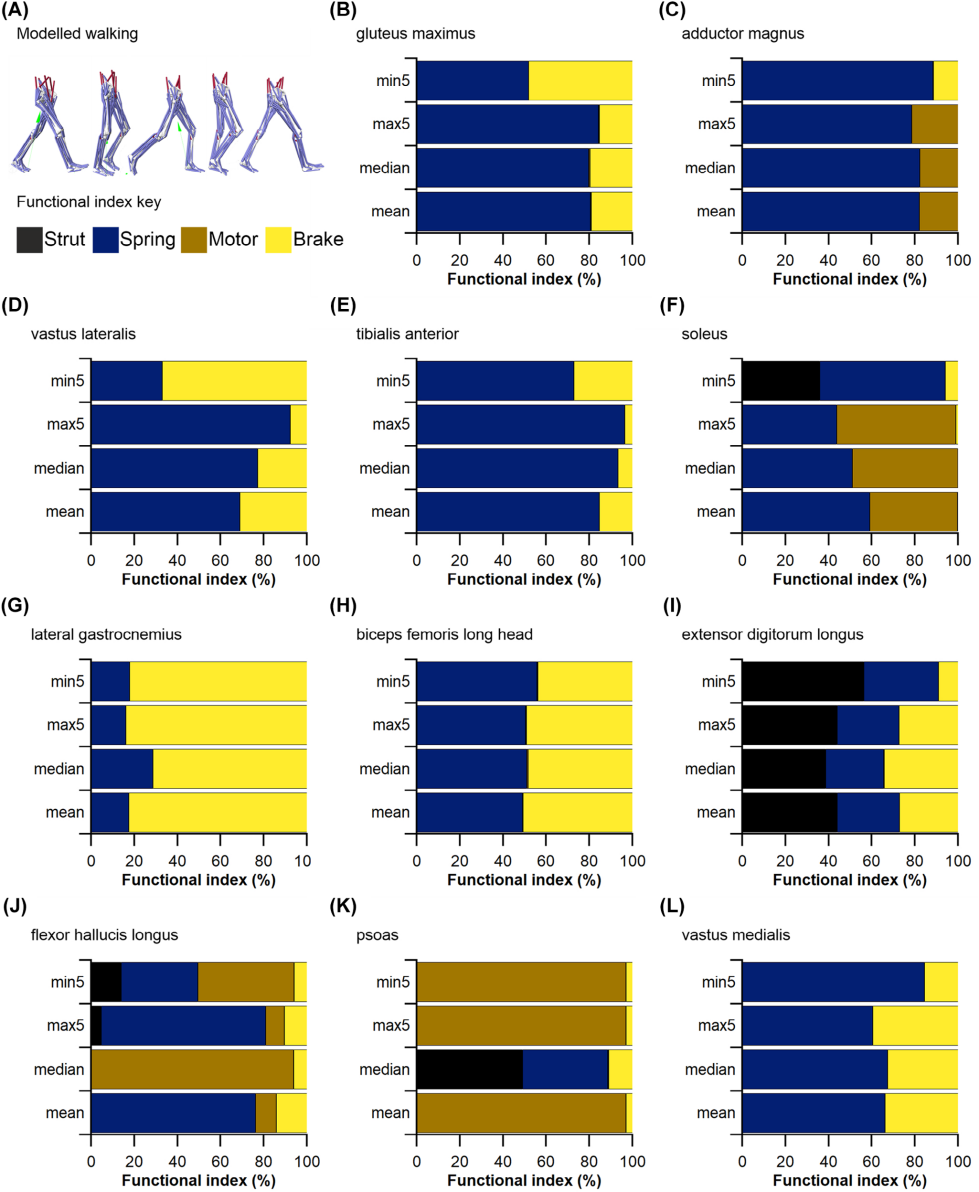
Functional implications of fibre length sample size as predicted by a musculoskeletal model and simulation of walking. In four different model interactions containing muscles with optimal fibre length and maximum force values informed by the full mean value (Model^mean^), median value (Model^median^), and the maximum (Model^max5^) and minimum (Model^min5^) possible values from random subsamples of 5 fibres, all muscles were characterised as acting as struts, springs, motors or brakes depending on the level and timing of force and power generation. In Model^mean^ and Model^median^, all muscles were informed by the appropriate values, while in Model^max5^ and Model^min5^, results are shown for only five muscles (B–F) informed by these properties, with the properties of all other muscles (e.g. G–L) determined by their Model^mean^ values. The vastus lateralis (D) and soleus (F) showed substantial changes in function relative to Model^mean^ due to reductions in fibre sample size (Model^min5^), while the flexor hallucis longus (J) showed a similarly large change in function in Model^min5^ despite no change in muscle force‐generating properties from Model^mean^.

**Fig. 13:**
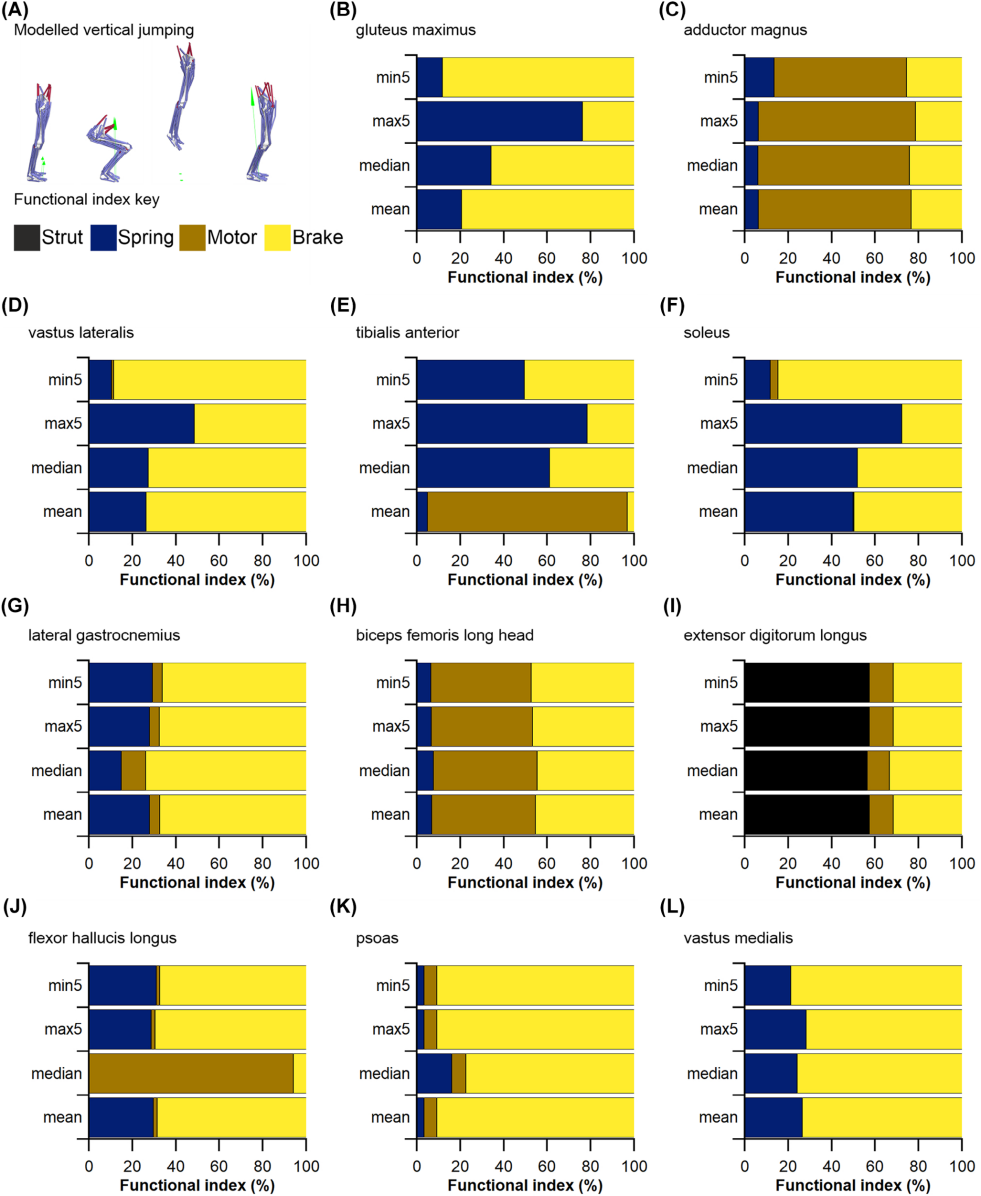
Functional implications of fibre length sample size as predicted by a musculoskeletal model and simulation of vertical jumping. In four different model interactions containing muscles with optimal fibre length and maximum force values informed by the full mean value (Model^mean^), median value (Model^median^), and the maximum (Model^max5^) and minimum (Model^min5^) possible values from random subsamples of 5 fibres, all muscles were characterised as acting as struts, springs, motors or brakes depending on the level and timing of force and power generation. In Model^mean^ and Model^median^, all muscles were informed by the appropriate values, while in Model^max5^ and Model^min5^, results are shown for only five muscles (B–F) informed by these properties, with the properties of all other muscles (e.g. G–L) determined by their Model^mean^ values. The tibialis anterior (E) and soleus (F) showed substantial changes in function relative to Model^mean^ due to reductions in fibre sample size (Model^min5^), while changes in function in muscles with unchanged force‐generating properties from Model^mean^ were much less than those seen during walking.

Potential errors in the quantification of muscle architecture due to (HYP1) low sample size (*n* fibres) and (HYP2) the use of mean instead of median values also have significant impacts on the quantitative aspects of muscle function derived from musculoskeletal simulations of dynamic movements (Figs 10, 11, 12, 13). Here, four different musculoskeletal models of one individual, which differed only in the muscle fibre lengths used to inform their muscle actuator force‐generating properties, were used to predict *in vivo* muscle fibre lengths, forces and mechanical work generation during walking (Fig. 10) and vertical jumping movements (Fig. 11). The root mean squared errors (RMSEs) of these outputs of the models which included MTU actuators with the maximum possible fibre length from a sample of 5 fibres (Model^max5^), the minimum possible fibre length from a sample of 5 fibres (Model^min5^) and the mean fibre lengths from the full sample of fibres (Model^mean^) were calculated relative to a model including MTU actuators with the median fibre lengths from the full sample of fibres (Model^median^) (Table S26). The largest errors relative to Model^median^ were seen in Model^min5^, where there were RMSEs in fibre length of 16.2 ± 5.2% (0.20 ± 0.07 mm) during walking (Fig. 10) and 13.7 ± 5% (0.19 ± 0.06 mm) during jumping (Fig. 11) when averaged across the tested muscles. Errors were substantially higher in terms of fibre force, with average errors of 401 ± 299% (337 ± 262 N) during walking (Fig. 10) and 266 ± 177% (1845 ± 2098 N) during jumping (Fig. 11). The muscle actuators that showed the largest error in Model^min5^ were Gmax, with errors in fibre force of 530% (579 N), 962% (797 N) and 502% (502 N) in the anterior, middle and posterior portions of this muscle respectively during walking (Fig. 10A; Table S26), and SOL, where there were errors of up to 537% (837 N) in fibre force during jumping (Fig. 11F; Table S26). The errors in both Model^max5^ and Model^mean^ were lower across all variables and all muscles. The largest error in the Model^max5^ outputs was a 54% (85 N) error in fibre force in SOL during jumping (Fig. 10F), while the largest error in the Model^mean^ outputs was a 121% (1526 N) error in fibre force in Gmax (mid portion) during jumping (Fig. 11A).

In terms of the functional indices, using median (Model^median^) instead of mean (Model^mean^) fibre lengths to inform muscle force‐generating properties results in different inferred fibre functions for several muscles during both walking (Fig. 12) and jumping (Fig. 13). During walking, these changes were most apparent in the flexor hallucis longus (FHL) and psoas (PMA) MTUs, where their fibres appeared to act as springs (equal positive and negative work; 76%) or motors (high force, positive work; 97%) respectively in Model^mean^, but motors (94%) or struts (high force, low work; 49%) respectively in Model^median^ (Fig. 12J, K). During jumping, the TA showed the greatest change in fibre function, with a primary motor function (92%) in Model^mean^ but a spring function (61%) in Model^median^ (Fig. 13E).

Similarly, the functions inferred from the Model^max5^ and Model^min5^ conditions also showed large changes relative to Model^mean^ through walking and jumping in the MTUs with altered force‐generating properties (Figs 12B–F, 13B–F) as well as the MTUs with unchanged properties (Figs 12G–L, 13G–L). For instance, the Gmax showed a shift from a spring‐like function (80%) to a spring/brake function (51%/48%) in Model^min5^ during walking (Fig. 12B), and from a brake function (high force, negative work; 79%) to a spring function (76%) in Model^max5^ during jumping (Fig. 13B). The SOL fibres also showed a large change in function in Model^min5^, with a switch from a spring/motor (59%/40%) to a strut/spring function (36%/58%) during walking (Fig. 12F), and from a spring/brake (49%/50%) to a primarily braking function (84%) in jumping (Fig. 13F). Interestingly, it was not solely the muscles in which the fibre length values were altered in the Model^min5^ and Model^max5^ conditions that showed substantial changes in functional roles, with the fibres of the FHL having a more motor‐like (9 to 44%) function in Model^min5^ during walking (Fig. 12J), and the vastus medialis (VM) becoming slightly more spring‐like (65 to 84%) (Fig. 12L), despite no change in their own force‐generating properties from Model^mean^. This demonstrates that even the qualitative functional role predicted for muscles with ‘accurate’ data (i.e. those provided by measuring a large number of fibre lengths) can be substantially affected by inaccurate architecture data in other muscles.

It should also be noted that measurements of muscle fibres entered into our models are influenced by other potential sources of error not considered fully here. In particular, this includes measurements of sarcomere length, which in this study were taken from previous work (Ward *et al*., 2009) and used to adjust the mean and median fibre lengths measured from DTI to an ‘optimal’ length. This was necessary as gathering *in vivo* sarcomere lengths from each muscle in each subject was outside the scope of this study. The use of these data was considered valid as they were gathered from tissue fixed in the anatomical position, which is how each subject was positioned during MR image acquisition. However, published sarcomere lengths vary both within and between previously published data sets, with Ward *et al*. (2009) reporting large standard deviations associated with their mean values, and Cutts (1988) reporting substantially different values despite specimens in both studies being fixed in the same anatomical position. Using the maximum mean ± standard deviation value from Ward *et al*. (2009) to optimise the fibre lengths reported here resulted in maximum errors of 17% in the VM MTU, while the maximum error introduced by using data from Cutts (1988) was 13% in the rectus femoris (RF) MTU (Table S27). However, it has been shown that sarcomere lengths vary considerably within a muscle, as well as among individuals, so eliminating this error even with primary individualised data is unlikely (Lichtwark *et al*., 2018). Ultimately, using previously published sarcomere length data to optimise subject‐specific fibre length data from DTI did introduce some degree of error into the derivation of the optimal fibre length values reported here. However, these errors were small compared to those potentially introduced by measuring a small initial sample size of fibres (up to 203% in the FDL).

It should also be noted that biomechanical models and simulations, as used here, provide estimates or approximations of skeletal muscle function during locomotor tasks. While the models used here were based on individualised muscle properties and musculoskeletal geometry, many simplifications remained, such as limited rotational and translational degrees of freedom at the joints, simplified muscle lines of action and generic human values for certain physiological properties (Charles *et al*., 2020). These simplifications will undoubtedly have introduced some error in our model output predictions (Figs 10, 11, 12, 13), but it is unlikely that this influenced the conclusions drawn from our models with respect to HYP3.

### (4) How do fibre sample size‐related errors impact upon higher‐level interpretations of muscle function across species?

**Fig. 14:**
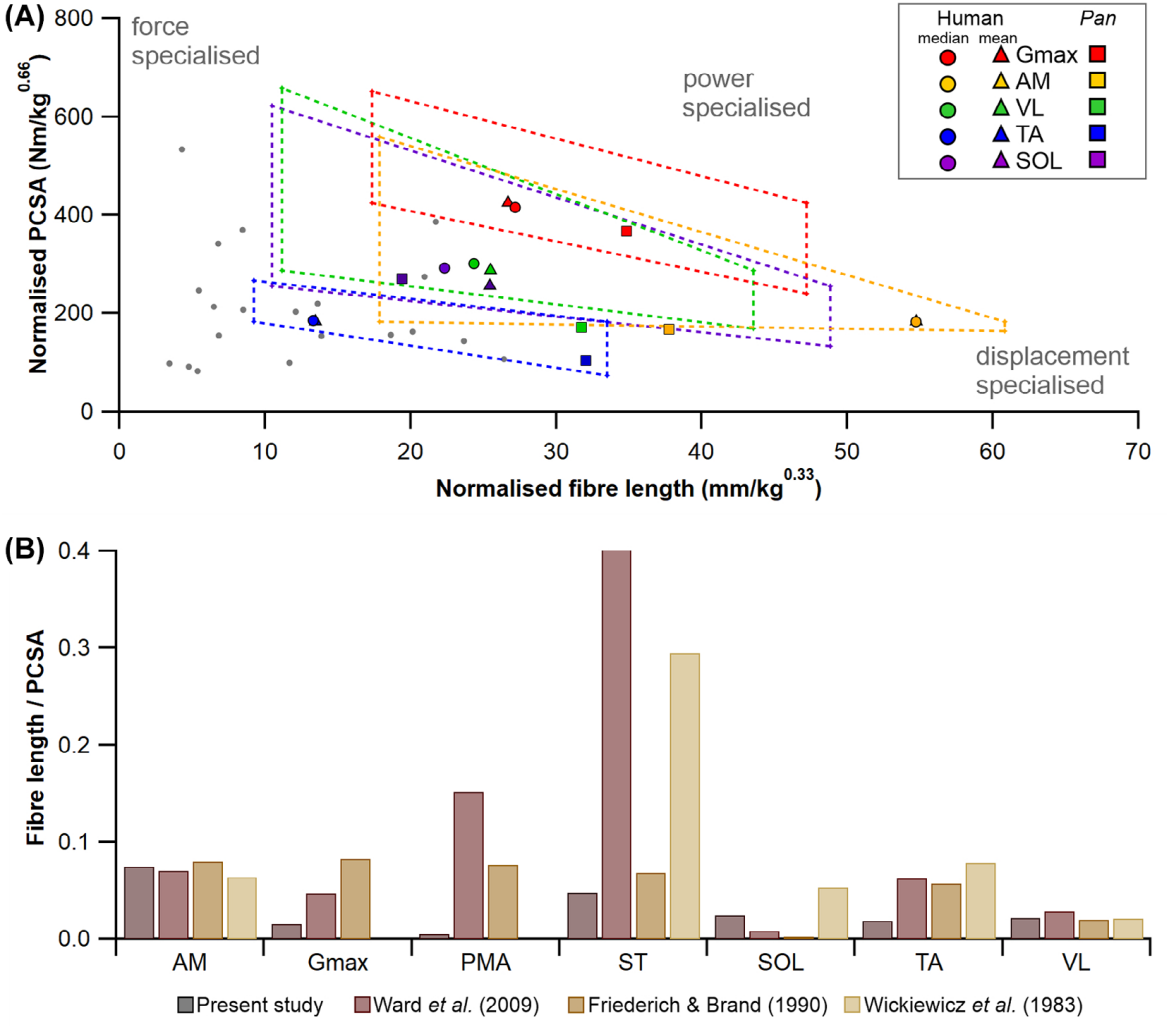
Interpretations of muscle function and specialisation based on fibre architecture. (A) Relationship between normalised muscle fibre length and normalised physiological cross‐sectional area (PCSA) across human (●, ▴) and chimpanzee (■) lower limb muscles. Median fibre lengths are in grey (●) with selected muscle‐specific data points highlighted in colour, and with a colour‐matched mean data point (▴). Dashed quadrilaterals depict potential error in fibre length and PCSA dependent on the number of fibres used to calculate fibre length. These uncertainties yield large potential error on inferences of muscle functional specialisations (inferred from the relationship between fibre length and PCSA), where random subsamples of 5 fibres can predict a muscle to either be displacement specialised or force specialised, depending on the mean value used. (B) Muscle fibre length to PCSA ratio in human muscles derived from data in this study and previously published work. The ratios of muscle fibre length to PCSA in selected muscles of the lower limb for the data presented here compared to those from previous studies highlight the range of functional behaviours inferred for certain muscles, particularly the semitendinosus (ST), which may be interpreted as resulting from measurements of only a small subset of fibres in past literature. AM, adductor magnus; Gmax, gluteus maximum; PMA, psoas; SM, semitendinosus; SOL, soleus; TA, tibialis anterior; VL, vastus lateralis.

Errors in the quantification of muscle architecture due to (HYP1) a low *n* fibres measured and (HYP2) the use of mean instead of median values also had a significant impact upon interpretations of muscle specialisation in humans *versus* chimpanzees (Fig. 14). When the full samples of fibres were used to calculate muscle properties, each muscle in which force‐generating properties were changed (Gmax, AM, VL, SOL and TA) showed different qualitative specialisations within human lower limbs (Fig. 14A). Gmax is recovered as the most power‐specialised lower limb muscle, capable of generating very high absolute forces across a relatively large working (length) range (Fig. 14A). Proportionally long fibres and a relatively small PCSA means that the AM is recovered as by far the strongest displacement‐specialist muscle, adapted to maintaining modest force output across a large working range (Fig. 14A). The TA shows little specialisation among surrounding lower limb muscles, having both modest PCSA and fibre length values, while both the VL and SOL are similar in showing a moderate degree of power‐specialisation (Fig. 14A).

However, the high variability in mean fibre lengths (and subsequently PCSA values) that are potentially possible when only measuring 5 fibres (Figs 6, 7, 8) means that an extremely broad continuum of specialisations (spanning the functional space occupied by a very large portion of all human lower limb muscles) become possible for all of these muscles (Fig. 14A). As a result, there is clearly significant potential qualitatively to misclassify the adaptive specialisation of muscles within a species both in absolute and relative terms when small fibre numbers are used. For example, at 5 fibres there is considerable potential to lose the displacement‐specialisation of AM, erroneously interpret TA, VL and SOL as displacement or force‐specialised muscles, and to recover any of Gmax, VL, SOL or AM as the most force, displacement or power‐specialised lower limb muscle in humans (Fig. 14A). In other words, the magnitude of potential inaccuracy seen at *n* = 5 fibres means that any combination of specialisation across all muscles might be recovered depending on the specific fibres measured.

Studies of muscle specialisation (Fig. 14) are common not only in zoological studies (Payne *et al*., 2005a; Allen *et al*., 2010) but also in palaeontology where the extant phylogenetic approach (Witmer, 1995) is used to identify transitions in muscle architecture and function that must have occurred in fossil lineages that bridge extant groups, such as human and non‐human great apes (Bramble & Lieberman, 2004) (human evolution) and birds and crocodilians (Bates & Schachner, 2012) (dinosaur evolution). In both contexts, differences in the location of homologous muscles within function plots (Fig. 14A) are often interpreted as functional adaptations that have evolved to facilitate different locomotor characteristics of the species under study (Payne *et al*., 2005a; Allen *et al*., 2010; Bates & Schachner, 2012). For example, in primatology and human evolution, such an approach has been used to hypothesise adaptive changes in muscle design that enabled the evolution of efficient upright walking and enhanced running ability in modern humans (Bramble & Lieberman, 2004; Carlson, 2006; Myatt *et al*., 2011). To optimise the efficiency of striding bipedalism, the muscle groups of the human lower limb have functional specialisations organised in a somewhat proximo‐distal gradient (Wickiewicz *et al*., 1983; Daley, Felix & Biewener, 2007; Ward *et al*., 2009), in which proximal muscle groups such as the hip extensors are thought to be more displacement specialised, with long parallel muscle fibres and relatively low PCSAs, and distal muscle groups such as the ankle plantarflexors having relatively shorter fibres and higher PCSA for more force specialisation. This gradient of muscle function is a feature of many cursorial mammals (Payne *et al*., 2005a; Williams *et al*., 2007a, 2008b), but is less strongly pronounced in animals using more flexed‐limb postures such as the primarily knuckle‐walking chimpanzee (*Pan troglodytes*) (O'Neill *et al*., 2013). There are therefore thought to be significant muscle architectural differences between the lower limb muscles of humans and *P. troglodytes* which highlight adaptations for different locomotor repertoires. Here we show the scale of these differences can be removed, exaggerated, or reversed by potential errors in functional inferences brought about by measuring small samples of muscle fibres (Fig. 14A). It is therefore clear that the large range of fibre lengths and PCSA values possible at low *n* fibre numbers (Figs 7, 8) creates significant potential for qualitatively incorrect interpretations of evolutionary adaptations in muscles across major functional transitions (Fig. 14).

The potential for significant quantitative and qualitative error in muscle function analysis at low *n* fibre numbers (Fig. 14) is not surprising given that high magnitudes of error in mean or median fibre lengths synchronously translate into large errors for muscle PCSA (e.g. 73% error in mean fibre length yielding 274% error in SOL; Figs 7, 8; Table S14). Indeed, this effect of fibre sample size may have resulted in some of the functional specialisations inferred here from muscle architecture alone to be different from those reported in previous studies (Wickiewicz *et al*., 1983; Friederich & Brand, 1990; Ward *et al*., 2009) (Fig. 14B). Functional specialisations of muscles have also been quantified using the *L*_f_:PCSA ratio, where a high value indicates a tendency for the muscle to be displacement specialised, and a low value suggesting it is force specialised. In previous studies of human lower limbs (where only 10–20 fibres were measured), muscles such as the PMA were predicted to be more suited for velocity in contraction (i.e. displacement specialised) than they were in this study, where they appeared more suited for force generation (Fig. 14B). Additionally, the data from Ward *et al*. (2009) and Wickiewicz *et al*. (1983) suggested that the semitendinosus (ST) muscle is a displacement specialist, whereas the data from Friederich & Brand (1990) (where up to 200 fibres were measured from each muscle) showed this muscle to be more of a force specialist, which is in line with the inferences recovered here (Fig. 14B). Of course, these discrepancies could be due to factors other than fibre number, such as anatomical variation, but the large range of possible fibre length values attainable from only a small sample of fibres suggests it is likely to be a primary cause behind variations in the data presented in previous muscle architecture studies.

### (5) Future perspectives

**Fig. 15:**
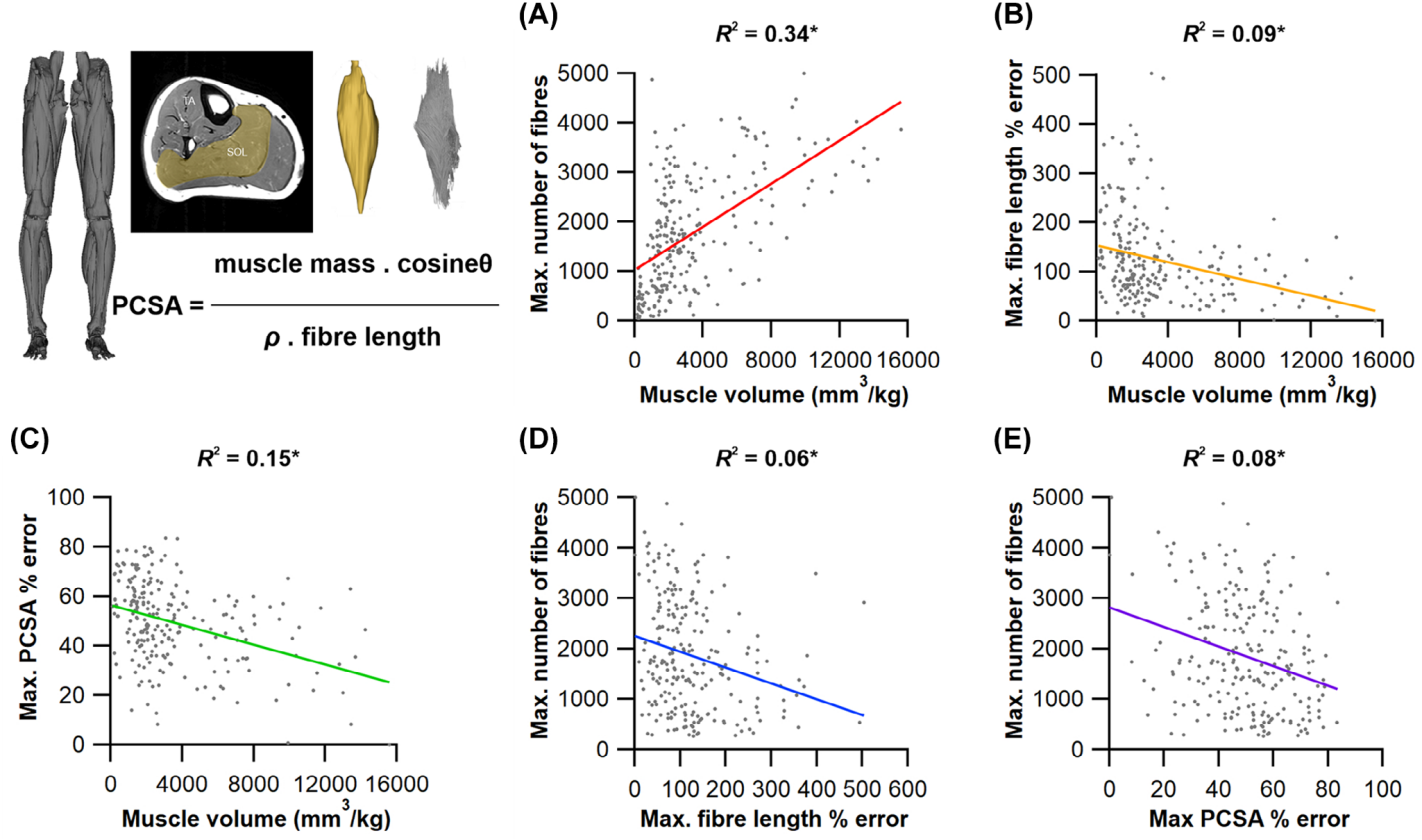
The relationships between muscle volume, maximum fibre number and percentage error in the lower limb muscles. (A) There was a moderately strong positive relationship between normalised muscle volume and the maximum number of fibres present, as measured using diffusion tensor imaging and deterministic fibre tractography. Neither of these metrics were strongly correlated with the maximum recovered percentage errors in either fibre length (B, D) or physiological cross‐sectional area (PCSA; C, E). This suggests that other factors, such as muscle architectural complexity, could instead explain the variation in fibre length, PCSA and contractile capacity errors seen here for the muscles of the lower limb. *P < 0.01. SOL, soleus.

We recover a clear tendency for certain muscles to show greater variation in mean and median *L*_f_ at small sample sizes than others. One possible explanation for this is systematic variation in error magnitude *versus* sample size is overall muscle size and total fibre number. As a preliminary assessment of size‐based variation in error, we investigated the statistical relationships between muscle volume, maximum fibre *n* and the maximum potential percentage errors in both *L*_f_ and PCSA in all lower limb muscle across all our subjects (Fig. 15). As expected, while the larger muscles of the lower limb tend to contain more fibres than the smaller muscles (*R* ^2^ = 0.32; Fig. 15B), there was little evidence for a relationship between either muscle volume or maximum fibre number and the % error in either *L*_f_ (*R* ^2^ = 0.09, 0.06, respectively; Fig. 15C, E) or PCSA (*R* ^2^ = 0.15, 0.08, respectively; Fig. 15D, F) resulting from a small fibre *n*.

It is therefore possible that this variation in recovered *L*_f_ and PCSA error within the muscles of the lower limb is due to differences in their fibre architectural arrangements. For instance, recent work using DTI has shown that the SOL muscle, which showed some of the largest *L*_f_ errors due to small fibre *n* (up to 78%) and was assumed here to be one homogeneous muscle, is actually composed of multiple compartments with substantially different fibre lengths and functional capabilities (Bolsterlee *et al*., 2018). This could also explain the relatively large *L*_f_ errors also seen in the AM muscle (also up to 78%), which was also assumed to be one whole muscle during the architecture measurements but is well known to include functionally distinct adductor and hamstring parts with separate insertions onto the femur.

**Fig. 16:**
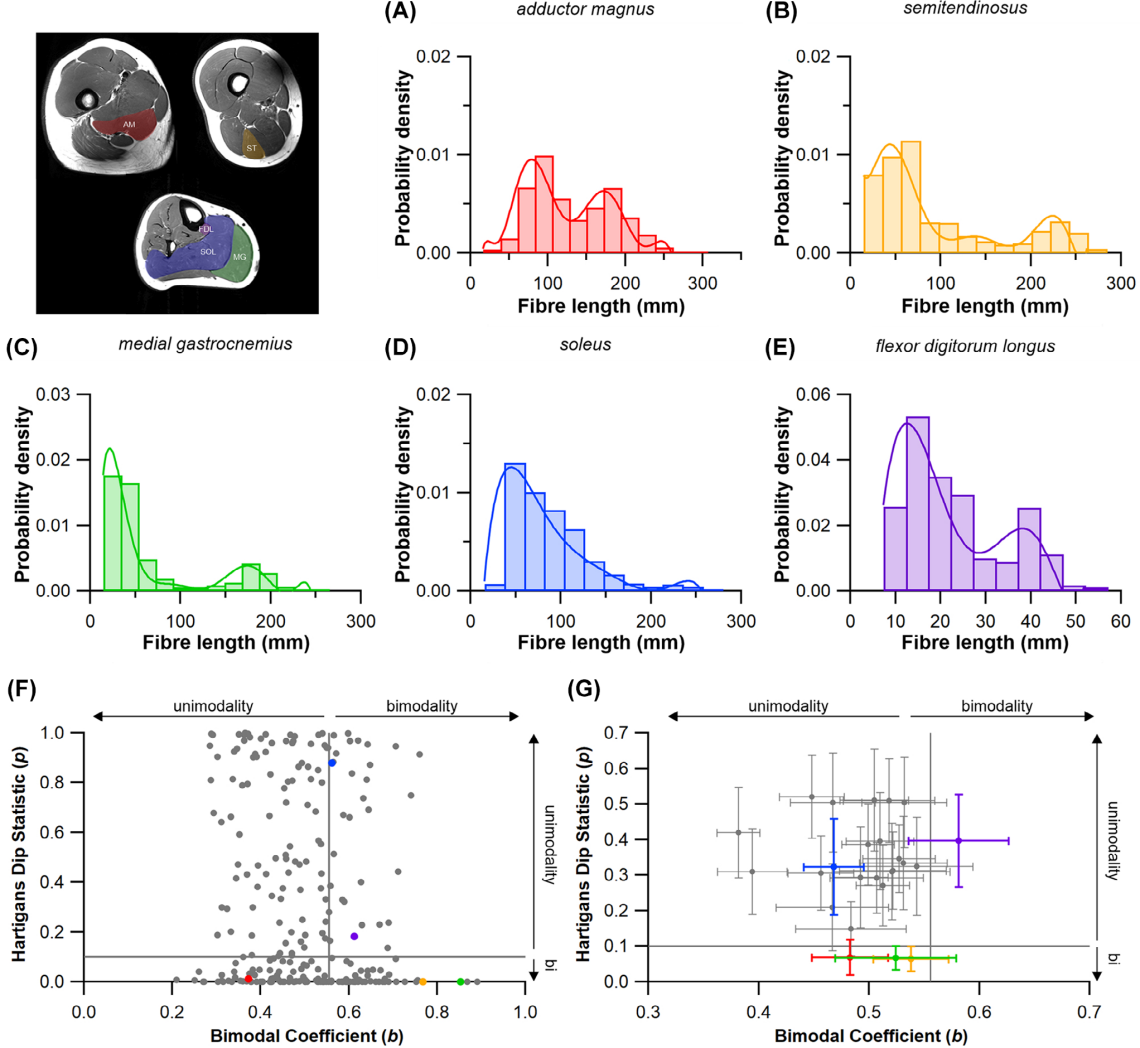
Statistical measures of bimodality in fibre length distributions. Data from all 250 muscles sampled were assessed using Hartigan's Dip statistic and the Binomial Coefficient equation to assess if the distribution of muscles fibres presented as a uni‐ or bimodal distribution. (A–E) Example distributions taken from the adductor magnus (A), semitendinosus (B), medial gastrocnemius (C), soleus (D) and flexor digitorum longus (E) highlight the variability in fibre distribution. (F) Of the 250 individual muscles sampled, 153 were considered bimodal in distribution. 77 were identified only by Hartigans Dip statistic, 30 were considered multi‐modal only by the Binomial Coefficient, with 46 considered multi‐modal by both statistical tests. (G) Presented as a muscle average across the 10 participants the adductor magnus (A, G), semitendinosus (B, G) and medial gastrocnemius (C, G) were classified as statistically bimodal by the Hartigans Dip Statistic only (*p*) while the flexor digitorum longus (E, G) was classified as bimodal by the Binomial Coefficient only. The soleus (D) was classified as unimodal by both statistical tests. AM, adductor magnus; FDL, flexor digitorum longus; MG, medial gastrocnemius; SOL, soleus; ST, semitendinosus.

Interestingly however, statistical analyses carried out here in association with HYP2 revealed that this compartmentalisation may be present in more muscles of the lower limb. In studying the distribution of fibre lengths throughout each muscle of each subject (250 muscles), 44% and 30% were identified as being multimodal in distribution using the Hartigan's Dip Statistic and Binomial Coefficient (BC) respectively (Fig. 16; see Appendix S1 for further details). This was most significant in the AM, ST, MG and FDL muscles amongst those studied here, with a potential error in *L*_f_ of up to 203% recovered from the FDL due to a small initial *n* (Table S14). This proposes interesting issues not only when predicting the functional behaviour of these muscles where whole muscle outputs are often informed by a single fibre length value, but also somewhat questions the conceptualisation of the muscle as a single actuator in general. Ultimately, the relatively low resolution (2.96 × 2.96 × 6.5 mm^3^) and large field of view (proximal aspect of the iliac crest to the dorsal aspect of the foot) of the DT images in this study [particularly compared to Bolsterlee *et al*. (2018) who used a resolution of 1.875 × 1.875 × 5 mm^3^ to image the lower leg only] prevented the accurate separation of the different compartments of these muscles here. However elucidating the extent of architectural compartmentalisation within skeletal muscle and the potentially disparate functions of these compartments represents an important area of future research. This could build on current studies by combining different imaging and analysis methods such as higher resolution diffusion tensor imaging, fibre tractography, functional *in vivo* muscle imaging (i.e. shear wave elastography; Vigotsky, Rouse & Lee, 2020), high‐density electromyography (Rojas‐Martinez *et al*., 2020) and musculoskeletal modelling fully to investigate the functional consequences of unique muscle architecture distributions within skeletal muscle.

## V CONCLUSIONS

1. Extrapolations between fibre architecture and performance underpin our understanding of how muscles function and how they are adapted to power specific motions within and across species (Fig. 1). Here we provide a synopsis of how this fibre to function paradigm has been applied to understand muscle design, performance and adaptation in animals. Our review highlights the widespread application of the fibre to function paradigm across a diverse breadth of biological disciplines but also reveals a potential and highly prevalent limitation running through past studies. Specifically, we find that quantification of muscle architectural properties is almost universally based on an extremely small number of fibre measurements. Despite the volume of research into muscle properties, across a diverse breadth of research disciplines, the fundamental assumption that a small proportion of fibre measurements can accurately represent the architectural properties of a muscle has never been quantitatively tested.
2. By combining DTI and deterministic fibre tractography (Bolsterlee *et al*., 2019; Charles *et al*., 2019a) we were able rapidly to generate a large number of fibre lengths for human lower limb muscles; more than 3500 in many muscles, which represents approximately two and a half times the highest number in any previous muscle study (Rosin & Nyakatura, 2017), and between 200 and 1666 times higher than the current standard observed in more than 80% of previous literature (Fig. 4A; Table S1). With this large data set were able to test, for the first time, the most basic assumptions commonly made when measuring muscle fibre lengths to calculate muscle force‐generating properties, which are subsequently used to understand muscle functional behaviour in both qualitative (comparative) and quantitative contexts following the fibre to function paradigm (Fig. 1C).
3. Through statistical subsampling simulations of our large fibre data sets, we demonstrate that the measurement of only a small number of fibres (*n* < 25) typical in previous studies may potentially realise extremely large errors in the characterisation of overall muscle architectural properties such as mean fibre length and physiological cross‐sectional area (Figs 6, 7, 8), which are key determinants of muscle force production and function.
4. Through dynamic musculoskeletal simulations of human walking and jumping, we demonstrate that the recovered errors in fibre architecture characterisation have significant implications for quantitative predictions of *in‐vivo* dynamics and muscle function within a species (Figs 10, 11, 12, 13).
5. By applying data subsampling simulations to comparisons of muscle function in humans and chimpanzees, we demonstrate that error magnitudes significantly impact both qualitative and quantitative assessment of muscle specialisation, potentially generating highly erroneous conclusions about the absolute and relative adaption of muscles across species and evolutionary transitions (Fig. 14).
6. These data and analyses demonstrate the importance of accurate fibre architecture measurements and the benefits of DTI and fibre tractography over more traditional dissection methods in allowing the relatively rapid and automated measurement of a large set of muscle fibres in order to predict muscle functional behaviour. Future developments of MR imaging and tractography, and integration with *in‐vivo* experimental measures and modelling approaches, will likely provide deeper insights into the functional consequences of complex and heterogenous muscle architecture distributions within skeletal muscle.
7. The level of accuracy or reliability required for any analysis depends on the goals of the experiment or the specific hypotheses being tested. The effects of the number of fibres analysed per muscle derived herein provide general guidance in this respect for a number of metrics in healthy mammalian skeletal muscle that could be used to guide power analyses in future studies, alongside other data sets where high fibre numbers have been measured. Ideally, future work in this area will formally incorporate sample size considerations into experimental design.

## Supporting information


**Appendix S1.** Additional information on methods.

**Table S1.** Details of the methods used in studies measuring vertebrate muscle architecture, specifically detailing the number of muscle fibres measured to obtain muscle fibre length.
**Table S2.** Study participant information.
**Tables S3–S12.** Fibre architecture and distributions of the individual study subjects.
**Table S13.** Mean and median fibre length (*L*_f_) and physiological cross‐sectional area (PCSA) values averaged across all 10 subjects.
**Table S14.** All‐subject averages in absolute and percentage differences in fibre length and physiological cross‐sectional area (PCSA) between the mean and median values of the full sample of fibres and the maximum and minimum possible mean and median values from random subsamples of 5 fibres.
**Tables S15–S24.** The percentage of randomly generated mean fibre length values in subsamples of 5, 10, 50, 100, 250, 500, 1000 and 2000 fibres falling in each ‘accuracy bin’ relative to the mean value from the full sample of >3000 fibres in 25 lower limb muscles from the individual study subjects.
**Table S25.** All‐subject averaged percentages of randomly generated mean fibre length values in subsample of 5, 10, 50, 100, 250, 500, 1000 and 2000 fibres falling in each ‘accuracy bin’ relative to the mean value from the full sample of >3000 fibres in 25 lower limb muscles, and these values averaged across all muscles for all subjects.
**Table S26.** Root mean squared errors (RMSEs) of fibre lengths and forces as predicted by musculoskeletal models containing mean (Model^mean^) fibre lengths as well as maximum (Model^max5^) and minimum (Model^min5)^ possible fibre lengths from samples of 5 fibres relative to a model containing median fibre lengths (Model^median^) during walking and vertical jumping.
**Table S27.** Variations in possible estimates of optimal fibre length possible from using different sarcomere length values from various literature sources.
