# Supplementary material for: From fibre to function: are we accurately representing muscle architecture and performance?
Source: Biol Rev Camb Philos Soc. 2022 Apr 7;97(4):1640–76. doi: 10.1111/brv.12856 (PMC9540431; doi:10.1111/brv.12856)
Supplement: Supplementary file 1 — Appendix S1. Additional information on methods. [file BRV-97-1640-s002.docx]

**SUPPORTING INFORMATION**

**From fibre to function: are we accurately representing muscle architecture and performance?**

James Charles^1,2¶^, Roger Kissane^2¶^, Tatjana Hoehfurtner^3^ & Karl T. Bates^2*^

^1^ Structure and Motion Lab, Comparative Biomedical Sciences, Royal Veterinary College, Hawkshead Lane, Hatfield, Hertfordshire, AL9 7TA

^2^Department of Musculoskeletal & Ageing Science, Institute of Life Course & Medical Sciences, University of Liverpool, The William Henry Duncan Building, 6 West Derby Street, Liverpool L7 8TX, UK;

^3^School of Life Sciences, University of Lincoln, Joseph Banks Laboratories, Green Lane, Lincoln LN6 7DL, UK

^¶^Joint first authors. These authors contributed equally to this work.

*Corresponding author E-mail: [k.t.bates@liverpool.ac.uk](mailto:k.t.bates@liverpool.ac.uk)

**Appendix S1. Additional information on methods**

**MRI data collection**

All MR images were obtained using a Siemens 3.0 T Prisma scanner (Siemens, Munich, Germany) with the following sequence parameters: T1-weighted anatomical TSE, voxel size 0.4395 × 0.4395 × 6.5 mm^3^, repetition time [TR] 700 ms, echo time [TE] 28 ms, number of slices = 36 per segment, number of signal averages (NSA) = 1; diffusion-weighted single-shot dual-refocusing spin-echo planar, voxel size 2.96 × 2.96 × 6.5 mm^3^, TR/TE 7900/67 ms, 12 direction diffusion gradients, *b* value = 0 & 400 s/mm^2^, strong fat suppression = spectral attenuated inversion recovery [SPAIR], number of slices = 36 per segment, NSA = 1, bandwidth 2350 Hz/pixel.

The T1- weighted MR images were digitally segmented in Mimics (Materialise, Leuven, Belgium) to create three-dimensional meshes of each muscle, while 5000 raw muscle fibre tracts were obtained from each muscle using deterministic fibre tractography in DSI studio (Yeh *et al.*, 2013). These fibre tracts were assumed to be functionally equivalent to muscle fibres (see Charles *et al*., 2019*a*,*b* for a discussion of this assumption). These raw tracts were then constrained in their length based on their corresponding volumetric mesh using an ‘anatomically constrained tractography’ post-processing toolbox (Bolsterlee *et al.*, 2017), which reduced the initial 5000 raw fibres down to an average of >3000 fibres per muscles. Mean muscle fibre length and pennation angle values were then obtained from these processed fibres.

For each muscle of the lower limb, these mean muscle fibre length values from DTI were used to calculate physiological cross-sectional area (PCSA^mean^) using the following formula:

$\mathrm{PCSA}^{\mathrm{mean}}= \frac{(V_{m} \times\cos\theta)}{L_{f}}$ , (S1)

where *V*_m_ is muscle volume, measured from 3D volumetric meshes of each muscle from T1 MR images, θ is mean pennation angle and *L*_f_ is optimal fibre length.

**Musculoskeletal modelling**

Maximum isometric force (*F*_max­_) for each muscle actuator was calculated by multiplying PCSA^mean^ by a generic value of maximum isometric stress in skeletal muscle [0.3 Nmm^2^; generally accepted to be valid for mammalian skeletal muscle (Hutchinson, 2004*a*; Medler, 2002; Zajac, 1989)]. Tendon slack lengths (*L*_ts_) were calculated through an optimisation algorithm accounting for minimum and maximum actuator and normalised fibre lengths (Manal & Buchanan, 2004). Muscles with broad origins were represented by multiple musculotendon unit (MTU) actuators in the model to recreate their functions better. The gluteus maximus (Gmax) was split into three separate MTUs (anterior, middle and posterior) and the adductor magnus (AM) muscle was split into two MTUs (hamstring and ischial part). In both muscles, the total *F*_max_ was split evenly between the MTU actuators, and the same *L*_f_ value was applied to each MTU. In the static optimisation simulations, residual and reserve actuators were appended to each unlocked degree of freedom to compensate for potential deficiencies in the muscle force-generating properties (Seth *et al.*, 2018).

The power generated from the fibres of each MTU in each model during walking and jumping was calculated by multiplying the instantaneous fibre forces and velocities predicted from the static optimisation simulations. From these values, the positive and negative mechanical work generated by the muscle fibres was calculated by integrating the positive and negative portions of these power curves. As described in previous studies (Lai *et al.*, 2019; Qiao & Jindrich, 2016), it was then possible from these work values to quantify the functional roles of the fibres of each MTU during walking and jumping through the calculation of four dimensionless functional indices: strut (high force generation but low amounts of work), spring (equal amounts of positive and negative work), motor (high amounts of positive work) and brake (high amounts of negative work); the cumulative percentage of which totalled 100%. Therefore, the functional index with the largest percentage could be considered the primary functional role of the fibres of a particular MTU actuator during the given movement. The strut indices (*I*_strut_) from the fibres of each MTU in each model condition were calculated as follows,

$I_{\mathrm{strut}}=\max\left( 1- \frac{\left( t_{\mathrm{FS}}\left( n+1 \right)- t_{\mathrm{FS}}\left( n \right) \right)\int_{t_{\mathrm{FS}}\left( n \right)}^{t_{\mathrm{FS}}\left( n+1 \right)} \left| P_{f} \right|dt}{l_{\mathrm{cha}}\int_{t_{\mathrm{FS}}\left( n \right)}^{t_{\mathrm{FS}}\left( n+1 \right)} \left| F_{f} \right|dt}, 0 \right) \times100\%$ (S2)

where *t*_FS_ is the time of foot strike, *n* is gait cycle number, *P*_f_ and *F*_f_ are fibre power and force respectively, and *l*_cha_ is a characteristic length change factor. The formula for *l*_cha_ is described in detail by Lai *et al.* (2019), but in brief it was optimised for each MTU to maximise its spring index relative to its tendon slack length.

A spring-like function of muscle fibres would involve energy absorption, or negative work, during fibre shortening and energy return, or positive work, during lengthening. Therefore, spring indices (*I*_spring_) were calculated as follows:

$I_{\mathrm{spring}}= \frac{2 \cdot min(\left| W_{l}^{-} \right|, \left| W_{s}^{+} \right|)}{\left| W_{\mathrm{tot}}^{-} \right|+\left| W_{\mathrm{tot}}^{+} \right|} \times100\%- I_{\mathrm{strut}}$ (S3)

where *W*_l_^–^ is the negative work when the fibres are lengthening, *W*_s_^+^ is the total positive work when the fibres are shortening, *W*_tot_^–^ is the total negative work and *W*_tot_^+^ is the total positive work. Motor indices (*I*_motor_) were calculated as follows:

$I_{\mathrm{motor}}= \frac{\left| W_{\mathrm{tot}}^{+} \right|-min(\left| W_{l}^{-} \right|,\left| W_{s}^{+} \right|)}{\left| W_{\mathrm{tot}}^{-} \right|+\left| W_{\mathrm{tot}}^{+} \right|} \times100\%- I_{\mathrm{strut}}$ (S4)

Brake indices (*I*_brake_) were calculated as follows:

$I_{\mathrm{brake}}= \frac{\left| W_{\mathrm{tot}}^{-} \right|-min(\left| W_{l}^{-} \right|,\left| W_{s}^{+} \right|)}{\left| W_{\mathrm{tot}}^{-} \right|+\left| W_{\mathrm{tot}}^{+} \right|} \times100\%- I_{\mathrm{strut}}$ (S5)

**Testing for multimodality in fibre length distributions (Figure 16 in main text)**

To test for potential multimodality in the distribution of fibre lengths within each muscle of each subject, fibre distributions were also assessed using both the Hartigan’s Dip Statistic (Freeman & Dale, 2013; Hartigan, 1985; Pfister *et al.*, 2013) and the Binomial coefficient (BC) (Freeman & Dale, 2013; Hartigan, 1985), which quantify multimodality in distinct ways. Hartigan’s Dip statistic was calculated in R (<https://www.r-project.org>) using the ‘dip’ function from the diptest package (R package version 0.75-7; see https://CRAN.R-project.org/package=diptest), generating a *p* value where *p*>0.05 is thought to be significantly bimodal in distribution (Freeman & Dale, 2013; Hartigan, 1985). The BC was calculated as:

$\mathrm{BC}= \frac{g^{2}+ 1}{k+ \frac{{3(n-1)}^{2}}{(n-2)(n-3)}}$ (S6)

where *g* is the skewness of the distribution, *k* is the excess kurtosis and *n* is the sample number. A BC >0.555 suggests that a distribution is binomial (Freeman & Dale, 2013; Hartigan, 1985; Pfister *et al.*, 2013).
